# Study on differentially expressed genes related to defoliation traits in two alfalfa varieties based on RNA-Seq

**DOI:** 10.1186/s12864-018-5180-1

**Published:** 2018-11-07

**Authors:** Qiming Cheng, Shiqie Bai, Gentu Ge, Ping Li, Liying Liu, Chengdong Zhang, Yushan Jia

**Affiliations:** 1 College of Grassland Resources and Environment, Key Laboratory of Forage Cultivation, Processing and High Efficient Utilization of the Ministry of Agriculture and Key Laboratory of Grassland Resources of the Ministry of Education, Inner Mongolia Agricultural University, Hohhot, 010011 China; 2 Sichuan Academy of Grassland Sciences, Chengdu, 611731 China; 3 Inner Mongolia Academy of Forestry Science, Hohhot, 010010 China; 4 School of Biotechnology and Biomolecular Sciences, University of New South Wales, Randwick, NSW 2052 Australia

**Keywords:** Alfalfa, Transcriptome, Differentially expressed genes (DEGs), Defoliation

## Abstract

**Background:**

Alfalfa (*Medicago sativa*) is a widely cultivated, essential commercial forage crop. The protein content in its leaves is the critical factor in determining the quality of alfalfa. Thus far, the understanding of the molecular mechanism of alfalfa defoliation traits remains unclear. The transcriptome database created by RNA-Seq is used to identify critical genes related to defoliation traits.

**Results:**

In this study, we sequenced the transcriptomes of the Zhungeer variety (with easy leaf abscission) and WL319HQ variety (without easy leaf abscission). Among the identified 66,734 unigenes, 706 differentially expressed genes (DEGs) upregulated, and 392 unigenes downregulated in the Zhungeer vs WL319HQ leaf. KEGG pathway annotations showed that 8,414 unigenes were annotated to 87 pathways and contained 281 DEGs. Six DEGs belonging to the “Carotenoid biosynthesis”, “Plant hormone signal transduction” and “Circadian rhythm-plant” pathways involved in defoliation traits were identified and validated by RT-qPCR analyses.

**Conclusions:**

This study used RNA-Seq to discover genes associated with defoliation traits between two alfalfa varieties. Our transcriptome data dramatically enriches alfalfa functional genomic studies. In addition, these data provide theoretical guidance for field production practice and genetic breeding, as well as references for future study of defoliation traits in alfalfa.

**Electronic supplementary material:**

The online version of this article (10.1186/s12864-018-5180-1) contains supplementary material, which is available to authorized users.

## Background

Alfalfa (*Medicago sativa* L.) is a member of the plant family *Fabaceae*. Alfalfa is widely distributed across the world and cultivated as a commercial forage crop due to its high protein, vitamin and mineral content [1, 2]. Mature alfalfa leaves are the primary crude protein storage organs (approximately 260 g kg^− 1^ dry basis), and protein content is a crucial indicator of the quality of alfalfa hay [3]. However, the leaves fall off very easily due to external pressure during harvest [4], and the resulting low-quality hay cannot satisfy livestock nutritional requirements.

The main factors in leaf senescence are endogenous abscisic acid (ABA) and ethylene (ETH) [5, 6]. ABA is a natural plant hormone identified in the 1960s, with roles in promoting plant organ shedding and seed germination [7, 8]. ABA is present in higher plant organs and tissues and especially abundant in mature and aging tissues or dormant organs. When plants are under biotic or abiotic stress, such as pathogen infection, drought or salinity, leaf senescence is induced by increasing ABA content [9–12]. ETH is another early-discovered plant hormone with function in the regulation of plant growth, such as the promotion of fruit ripening, acceleration of organ aging and shedding [13]. ETH is synthesized by conversion from methionine with enough oxygen, which occurs in various plant tissues and organs [14]. ETH synthesis is regulated by a variety of growth signals with tissue specificity at different development stages, and the accumulation of ETH can also stimulate further ETH production [15]. The content of ETH is significantly higher in the processes of fruit ripening, seed germination, and leaf and flower aging off [16, 17]. In addition, the synthesis of ETH is affected by a variety of external factors, such as mechanical damage, pathogen infection, low temperature, drought and salinity [18].

Previous studies showed that both ABA and ETH have the function of accelerating plant organ shedding. However, the physiological mechanisms are still unclear and remain controversial [19]. A study by Addicott [20] showed that endogenous ABA had positive effects on plant organ shedding, whereas field experiments failed, possibly because indoleacetic acid (IAA), gibberellin (GA) and cytokinin (CTK) in leaves have an adverse effect on ABA. Furthermore, the effect of introducing exogenous ABA on plant organ abscission was weaker than the effect of exogenous ETH. Osborne [21] concluded that ABA caused the premature senescence of organ cells, but that this may be due to increasing ETH content in plant organs that leads to organ shedding.

Transcriptome refers to a collection of all the transcription products under a specific physiological condition, including messenger RNA, ribosomal RNA, transport RNA and noncoding RNA [22]. Transcriptome sequencing research is essential for novel gene discovery, functional gene annotation, gene differential expression and the development of molecular markers [23–25]. Next Generation Sequencing (NGS) technology enables complete and rapid access to almost all transcript sequence information of a particular tissue or organ of a species and has been widely used in modal and nonmodel plant research [26–28]. To our knowledge, there have been no previous reports of research on DEGs related to defoliation traits in alfalfa varieties. This study aimed to construct a transcriptome database of two alfalfa leaf varieties in the early blooming stage. Based on DEGs, we aimed to identify the critical genes related to defoliation traits in the Zhungeer variety (characterized by easy leaf abscission) and the WL319HQ variety (without easy leaf abscission). The results of this study provide a theoretical basis for future alfalfa molecular breeding and provide a reference for subsequent study of the transcription of alfalfa leaves.

## Results

### De novo assembly transcriptome

**Fig. 1:**
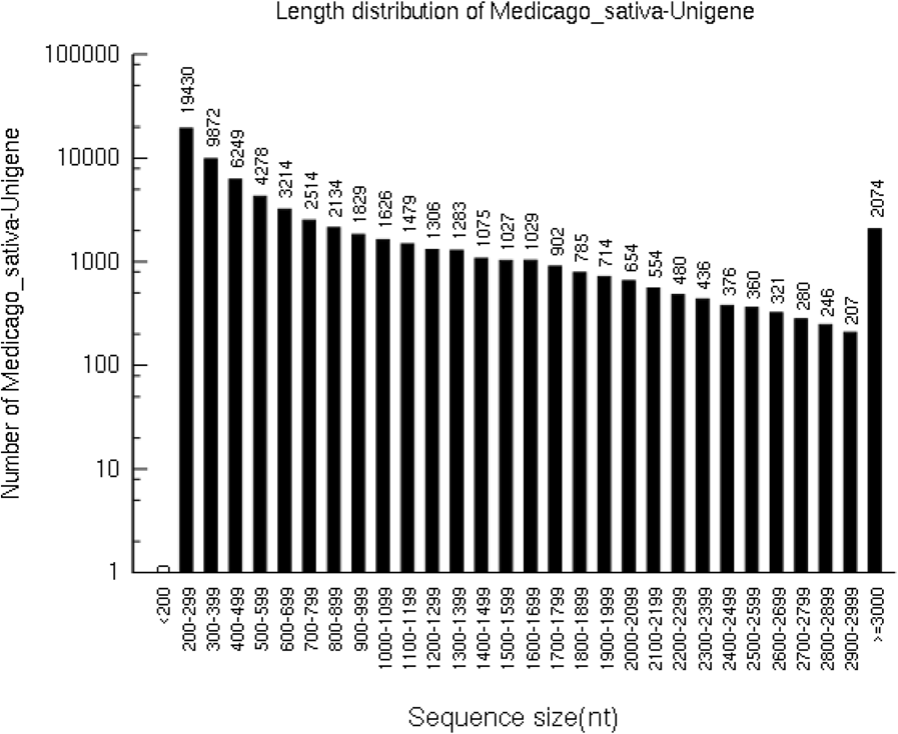
The size distribution of *M. sativa* unigenes. The abscissa is the length of the assembled unigenes from 200 nt to ≥3000 nt, and the ordinate is the number of unigenes of the corresponding length

Six libraries of the total RNA extracted from the leaves of Zhungeer and WL319HQ varieties at the early flowering stage were constructed for high-throughput sequencing. A total of 261,775,004 clean reads with a total of 39,202,954,927 nucleotides (nt) were obtained from the six sequencing libraries. Trinity method assembled a total of 66,734 unigenes with an N50 of 1496 nt. The maximum, minimum and average lengths were 143,789 nt, 201 nt and 869 nt, respectively (Fig. 1).

### Gene annotation

**Fig. 2:**
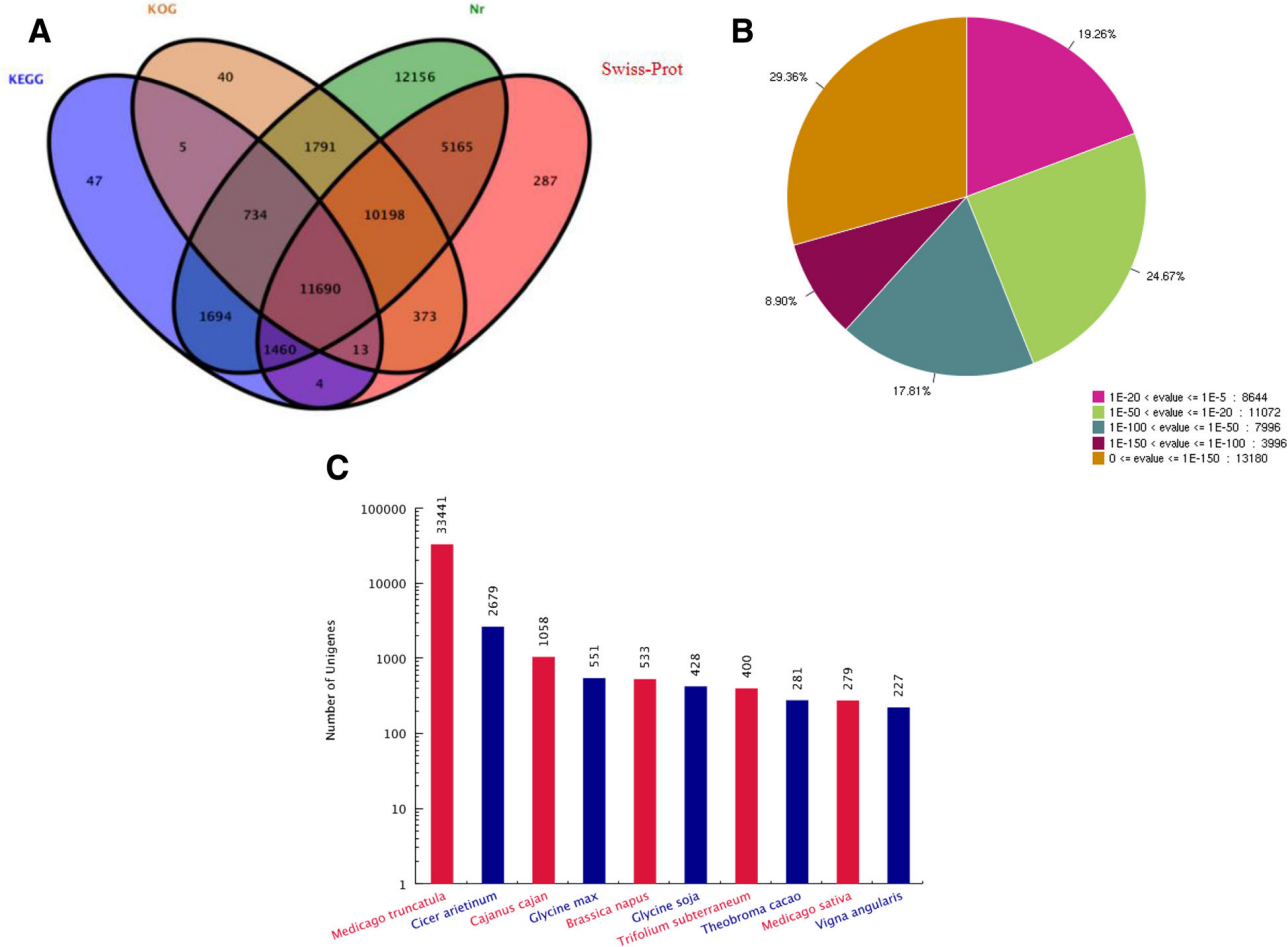
Homology search of *M. sativa* unigenes. **a** Venn diagram of unigene numbers annotated by BLASTx with a cut-off E-value of 1 × 10^− 5^ against protein databases. The numbers in the circles indicate the number of unigenes annotated by single or multiple databases. **b** E-value distribution of the top BLASTx hits against the Nr database. **c** Ten top BLASTx hits of homologous sequences for the species distribution

A total of 45,657 unigenes (68.42% of total 66,734 unigenes) were annotated against Nr, Swiss-Prot, KOG and KEGG databases using BLASTx (E-value < 1 × 10^− 5^). Among them, 42,888 (67.26%), 29,190 (43.74%), 24,844 (37.23%) and 15,647 (23.45%) unigenes were annotated to the Nr, Swiss-Prot, KOG and KEGG databases, respectively (Fig. 2a). According to the Nr database, 19.26% of unigenes showed homology (1 x E^− 20^ < E-value ≤1 x E^− 5^), 42.48% of unigenes showed strong homology (1 x E^− 100^ < E-value ≤1E^− 20^), and the remaining of 38.26% of unigenes had very strong homology (E-value ≤1E^− 100^) (Fig. 2b). For the species distribution of the top BLAST hits, 3,441 unigenes matched to the homologous sequences of *Medicago truncatula*, while 2,679, 1,058 and 551 unigenes matched to the homologous sequences of *Cicer arietinum*, *Cajanus cajan* and *Glycine max*, respectively (Fig. 2c).

### Identification and analysis of DEGs

**Fig. 3:**
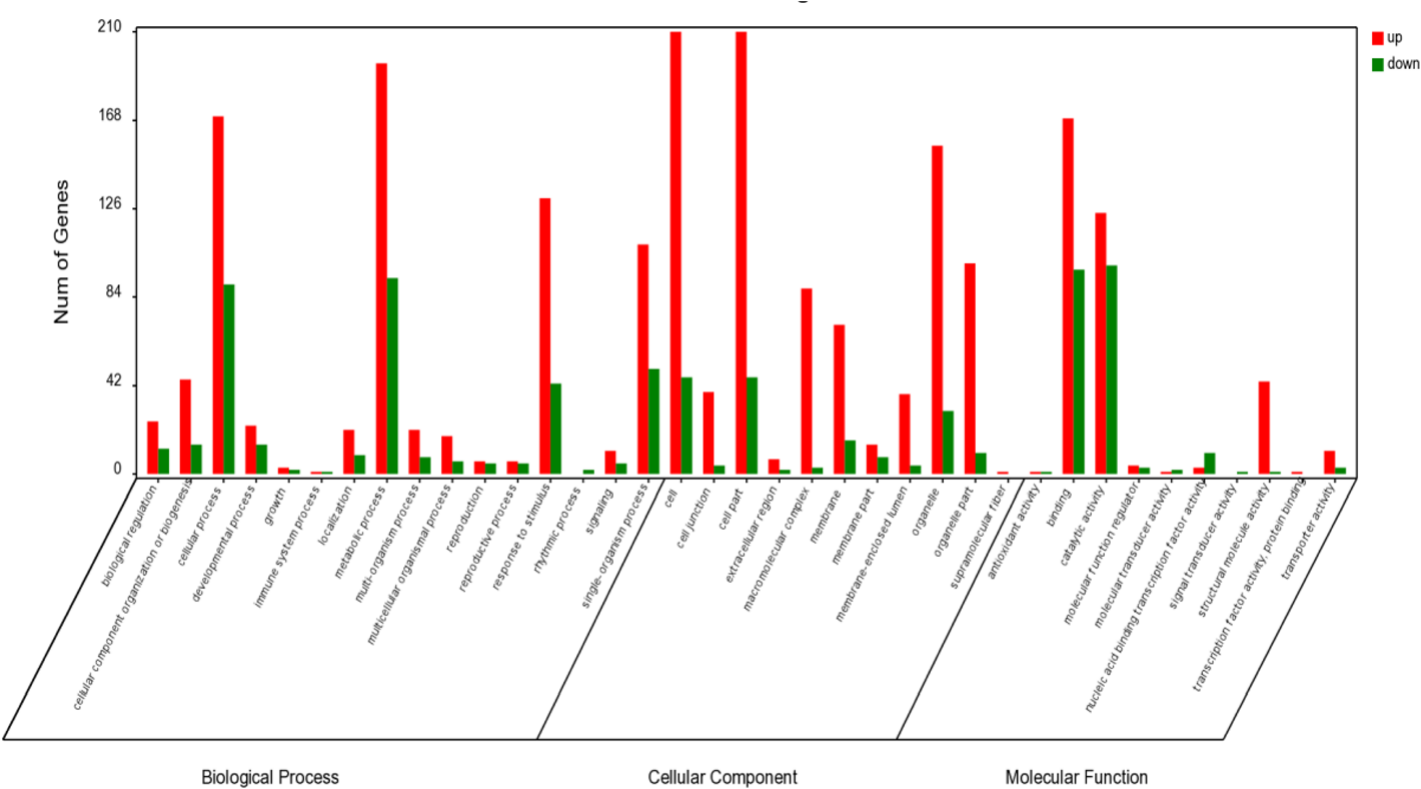
GO classification of assembled unigenes. A total of 17,156 unigenes were categorized into three main categories: biological process, cellular component and molecular function

Using the Nr annotation results, Blast2GO software was used to analyze GO functional annotations of unigenes, and WEGO software was used to perform the functional classification of unigenes. A total of 1,098 significant DEGs were assigned to one or more ontologies by the standard of |log2FC| > 1 and FDR < 0.05 (Additional file 1: Table S1; Additional file 2: Table S2; Fig. 3), 706 unigenes were upregulated, and the other 392 unigenes were downregulated (Zhungeer vs WL319HQ). The significant DEGs were used for the subsequent analysis.

In this group, “biological process”, “metabolic process”, “cellular process”, “response to stimulus” and “single-organism process” were the most frequent terms and contained 288, 260, 174 and 159 unigenes, respectively. For “metabolic process”, there were 195 upregulation unigenes and 93 downregulation unigenes. For “cellular process”, there were 170 upregulation unigenes and 90 downregulation unigenes. For “response to stimulus”, there were 131 upregulation unigenes and 43 downregulation unigenes. For “single-organism process”, there were 109 upregulation unigenes and 50 downregulation unigenes, whereas “immune system process” (two unigenes) and “rhythmic process” (two unigenes) were infrequent. Under the “cellular component” group, most of those were classified into “cell” (210 upregulation unigenes, 46 downregulation unigenes), “cell part” (210 upregulation unigenes, 46 downregulation unigenes), and “organelle” (156 upregulation unigenes, 30 downregulation unigenes). For the “molecular function” group, “binding” (169 upregulation unigenes, 97 downregulation unigenes) and “catalytic activity” (124 upregulation unigenes, 99 downregulation unigenes) were the most abundant subcategories.

### KEGG pathway enrichment analysis

A total of 8414 unigenes were assigned to 87 KEGG pathways (Additional file 3: Table S3). The 15 top KEGG pathways with the highest representation of DEGs were: “Ribosome” (ko03010), “Spliceosome” (ko03040), “Protein processing in endoplasmic reticulum” (ko0414), “Carbon metabolism” (ko01200), “Endocytosis” (ko04144), “Biosynthesis of amino acids” (ko01230), “RNA transport” (ko03013), “Plant-pathogen interaction” (ko04626), “Starch and sucrose metabolism” (ko00500), “Oxidative phosphorylation” (ko00190), “Nucleotide excision repair” (ko03420), “Plant hormone signal transduction” (ko04075), “DNA replication” (ko0303), “Ubiquitin-mediated proteolysis” (ko04120), and “Homologous recombination” (ko03440). 622 (14.58%) unigenes and 64 DEGs accounting for 22.78% of 281 DEGs were annotated to the “Ribosome” pathway.

In the 87 KEGG pathways, the DEGs related to direct or indirect effects on the content of ABA and ETH were predicted. In the “Plant hormone signal transduction” pathway (ko04075), 294 unigenes with 6 DEGs were annotated. In the “Circadian rhythm-plant” pathway (ko04712), 61 unigenes with 4 DEGs were annotated. In addition, the “Carotenoid biosynthesis” pathway (ko00906) contained 44 unigenes with 1 DEG crucial for ABA synthesis, which was annotated.

### Real-time quantitative PCR analysis of DEGs related to defoliation

**Fig. 4:**
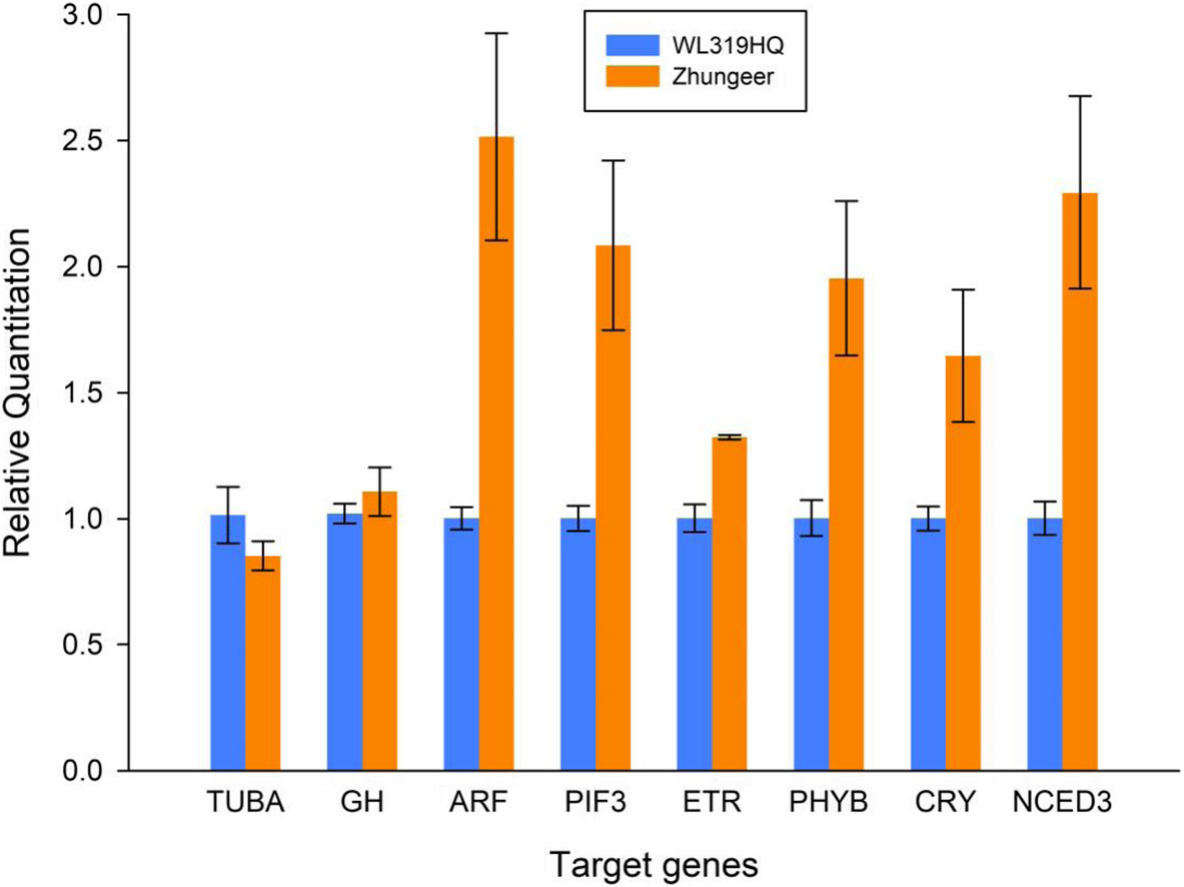
The relative expression level of DEGs involved in “Plant hormone signal transduction”, “Circadian rhythm-plant” and “Carotenoid biosynthesis” pathways revealed by RT-qPCR analysis. *TUBA*: Tubulin alpha, no significant difference (*P* = 0.165); *GH*: indole-3-acetic acid-amido synthetase, no significant difference (*P* = 0.246); *ARF*: Auxin response factor, significant difference (*P* = 0.033); *PIF3*: Phytochrome interacting factor 3, significant difference (*P* = 0.043); *ETR*: Ethylene receptor protein, significant difference (*P* = 0.014); *PHYB*: Phytochrome B, significant difference (*P* = 0.047); *CRY*: Cryptochrome, significant difference (*P* = 0.049); *NCED3*: 9-cis-epoxycarotenoid dioxygenase 3, significant difference (*P* = 0.04)

To test the reliability of transcriptome sequencing data in the three pathways, Unigene0044746 (Auxin response factor, *ARF*), Unigene0002039 (Phytochrome interacting factor 3, *PIF3*), Unigene0027311 (Ethylene receptor protein, *ETR*), Unigene0053251 (Phytochrome B, *PHYB*), Unigene0053032 (Cryptochrome, *CRY*) and Unigene0014585 (9-cis-epoxycarotenoid dioxygenase 3, *NCED3*) (Additional file 4: Table S4) were selected for One-Step RT-qPCR analysis. In addition to DEGs, Unigene0030464 (Tubulin alpha, *TUBA*) and Unigene0032887 (indole-3-acetic acid-amido synthetase, *GH*) were selected in the RT-qPCR analysis as unaffected genes. The alfalfa *GAPDH* gene was used as the endogenous reference. The significant difference of RT-qPCR data between two varieties was analyzed by t-test, and the expression patterns revealed by RT-qPCR analysis were similar to those revealed by transcriptome sequencing for the same genes (Fig. 4).

## Discussion

ABA, ETH, jasmonic acid, salicylic acid, CTK and auxin are the primary phytohormones that regulate plant senescence [29]. When plants are under environmental stress, such as drought, salinity or low temperature, the leaves will accelerate senescence and abscission due to the elevation of ABA concentration [11, 30]. In addition, ETH stimulates the synthesis of cellulase and controls the release of cellulase from protoplasts into the cell wall, which promotes cell wall degradation and leaf abscission [5]. Many studies reported that ETH levels would rise if plant tissues were damaged by mechanical force [4, 31, 32]. Therefore, the abscission of alfalfa leaves may be due to mechanical damage to plant tissues during harvesting, resulting in elevation of ETH concentration in leaf tissues. The mechanism by which phytohormone interactions promote the abscission of leaves is not well understood and is difficult to study [33].

In higher plants, xanthoxin is not only the synthesis precursor of ABA but also a metabolic intermediate of carotenoid catabolism [34]. The RNA-Seq data revealed that the alfalfa 9-cis-epoxycarotenoid dioxygenase 3 (*NCED3*) gene differentially expressed between WL319HQ and Zhungeer varieties. The RT-qPCR analysis showed that the transcription of the Zhungeer variety was approximately 2.3 times higher than the WL319HQ variety. *NCED* is the enzyme that catalyzes the cleavage reaction of 9-*cis-*epoxycarotenoids to produce xanthoxin and is the rate-limiting enzyme of ABA biosynthesis in higher plants [35–37]. *NCED* has been identified in maize, kidney bean, tomato and *Arabidopsis thaliana* [4, 35, 36, 38, 39]. *A. thaliana NCED* and its role in ABA biosynthesis are the most well-studied [37]. Overexpression of *A. thaliana NCED3* resulted in a significantly higher level of endogenous ABA in the plant [40], and similar results were obtained in transgenic tobacco to overexpress the tomato *NCED1* gene [38].

The “Plant hormone signal transduction” (ko04075) pathway plays a vital role in the metabolism of phytohormones in higher plants. In the RNA-Seq data, the expression level of the ethylene receptor protein (*ETR*) gene in Zhungeer variety was approximately 1.4 times higher than the WL319HQ variety. *ETR* is a protein that initiates the ETH signal transduction pathway by binding the ETH and relaying the ETH signal to constitutive triple response 1 (*CTR1*) Raf-like protein kinase [41]. The high-affinity binding activity has been confirmed in *A. thaliana*, as well as the tomato [42–44]. In the present study, the *ETR* gene was downregulated in WL319HQ compared to the Zhungeer variety, indicating that Zhungeer is more likely to accumulate ETH than the WL319HQ variety.

Auxin response factors (*ARF*) are transcription factors that bind specifically to the DNA sequence 5’-TGTCTC-3′ found in auxin-responsive promoter elements (AuxREs). Although *ARF* genes are not involved in leaf senescence directly, they act as transcriptional activators or repressors and therefore promote flowering, stamen development, floral organ abscission and fruit dehiscence [45]. Study of the *A. thaliana ARF1* and *ARF2* mutant has shown that *ARF2* may affect plant tissue shedding [46]. Nagpal et al. [47] found that the *A. thaliana ARF6* gene promotes flower organ maturation and senescence. In addition, *NPH4/ARF7* and *ARF19* not only enhance the *ARF2* phenotype but also induce leaf senescence [46]. The *ARF* gene was significantly downregulated in alfalfa leaves in the WL319HQ variety.

Plant circadian rhythm is a hereditary physiological regulatory mechanism that synchronizes with circadian alternation in the plant. The response of plants to environmental factors mainly depends on the hormone regulatory network, including CTK, IAA, ABA and GA [48]. Circadian rhythm plays an essential role in the plant hormonal regulation pathway [49, 50]. At the same time, the plant hormone signaling network also acts on circadian rhythm to transmit different metabolic signals and environmental signals to the endogenous circadian rhythm system, thus forming a complex regulatory network [51, 52]. In this study, three DEGs involved in the plant circadian rhythm pathway were identified that may affect the regulation of ABA and ETH. Compared with Zhungeer, the *PHYB* (− 1.775-fold), *PIF3* (− 3.715-fold) and *CRY* (− 1.952) gene of WL319HQ were downregulated. *PIF3* plays a critical role not only in light and temperature-mediated environmental signaling but also in the signaling of ABA and ETH. Light activates the phytochrome pathway to induce the degradation of *PIF3* and promotes the accumulation of carotenoids, which may lead to an increase in ABA content.

In summary, it is likely that the Zhungeer alfalfa variety is more susceptible to abscission than the WL319HQ variety due to higher levels of plant hormone accumulation. Higher expression of *NCED3* and *PIF3* genes caused increasing ABA content, which may be a reason why the Zhungeer variety is more likely to defoliate. In addition, upregulation of *ETR* and *ARF* expression affects ETH; auxin accumulation is another indirect evidence as well.

## Conclusion

This study is the first to use RNA-Seq to identify the defoliation traits between alfalfa varieties. It is essential to understand the physiological effects of various hormones on plants, the interaction between hormones and their relationship with the agricultural production environment. Our transcriptome data dramatically enriches alfalfa genome research. This research will provide theoretical guidance for field production practice and genetic breeding and provides a reference for the future study of defoliation traits in alfalfa.

## Methods

### Sample collection

We selected two varieties of the tetraploid *M. sativa*: the Zhungeer variety that has easy leaf abscission and the WL319HQ variety that is not characterized by easy leaf abscission. Both varieties were grown in experimental fields located in Baotou City, Inner Mongolia, China (110°37′E, 40°5’N). Leaves of the Zhungeer and WL319HQ varieties were collected from two-year-old plants at the early flowering stage in June. All samples were immediately frozen in liquid nitrogen and stored at − 80 °C for future RNA extraction.

### RNA extraction and RNA-Seq library construction

Total RNA was extracted from three biological replicates of the leaves of each *M. sativa* variety using the RNeasy Plant Mini Kit (Qiagen, Germany), following the manufacturer’s protocol. The integrity of RNA samples was measured by Agilent 2100 Bioanalyzer (Agilent Technologies, USA). The Poly (A) mRNA was enriched by NEBNext Oligo(dT)_25_ beads (NEB, USA) from 50 μl total RNA. Then, the enriched mRNA was constructed to a cDNA library by NEBNext Ultra RNA Library Prep Kit for Illumina (NEB, USA), following the manufacturer’s protocol.

### Sequencing and raw reads filtering

The transcriptome sequencing was performed using an Illumina HiSeq 4000 sequencing platform with paired-end 150 bp (PE150) sequencing strategy at Gene Denovo Biotechnology Co. (Guangzhou, China). Raw reads obtained from the sequencing that may affect the following assembly and analysis were removed, including reads with adapters, reads containing more than 10% unknown nucleotides and low-quality reads containing more than 50% low-quality bases (Q-values ≤10).

### De novo transcriptome assembly and unigenes detection

Since *M. sativa* genome information was not previously available, full-length transcriptome de novo assembly was carried out using the reference genome independent Trinity method [53]. Trinity is a software package consisting of three modules: Inchworm, Chrysalis and Butterfly. First, Inchworm assembled clean reads to a collection of linear contigs by the greedy k-mer method. Second, the related minimal overlapping contigs created by Inchworm were clustered into connected components by Chrysalis, and a de Bruijn graph was constructed for each cluster of related contigs. Finally, Butterfly analyzed the paths from the de Bruijn graphs that were taken by reads and pairs of reads, and the complete transcripts and unigenes sets were obtained from the outputs [54].

### Unigenes annotation

The unigenes were annotated using the BLASTx program (http://www.ncbi.nlm.nih.gov/BLAST/) with an E-value threshold of 1 × 10^− 5^ to Nr (NCBI nonredundant protein) database (http://www.ncbi.nlm.nih.gov), the Swiss-Prot (A manually annotated and reviewed protein sequence database) protein database (http://www.expasy.ch/sprot), the KEGG (Kyoto Encyclopedia of Genes and Genomes) database (http://www.genome.jp/kegg) and the KOG (Clusters of Orthologous Groups of proteins) database (http://www.genome.jp/kegg/ko.html). Protein functional annotations were obtained according to the best alignment results.

### Analysis of DEGs

Gene expression analysis of unigenes was performed and normalized by RPKM (reads per kb per million reads). The edgeR package (http://www.r-project.org/) was used to identify DEGs across samples or groups. Genes with a fold change ≥2 and a false discovery rate (FDR) < 0.05 were considered significant DEGs. DEGs were further subjected to GO functional enrichment analysis and KEGG pathway analysis.

### Real-time quantitative PCR analysis

One-Step Real-time quantitative PCR (RT-qPCR) was carried out to confirm the RNA-Seq data from the extracted *M. sativa* leaves’ total RNA. The experiments were performed using One-Step TB Green PrimeScript RT-PCR Kit II (Takara, Japan) on an ABI 7500 system (Applied Biosystems, USA). Primers for One-Step RT-qPCR were designed using Primer Premier 5.0 software (Premier, Canada). The alfalfa GAPDH gene was selected as the endogenous reference [55]. All the primers were synthesized by Invitrogen (Beijing, China) and are listed in Additional file 5: Table S5. A 20 μl reaction mix was set up containing 50 ng total RNA and 0.4 μM of each primer. The One-Step RT-qPCR analysis was performed on the extracted total RNA in three biological replicates of the leaves of each *M. sativa* variety, and each RNA sample was carried out in technical triplicate. The program was set at 42 °C for 5 min then 95 °C for 10 s, followed by 40 cycles of 95 °C for 5 s then 60 °C for 34 s. The final melting curve analysis was set at 95 °C for 15 s, 60 °C for 1 min and 95 °C for 15 s. The relative expression changes of the endogenous reference and tested genes were analyzed by the 2 ^- △△ CT^ method [56].

### Statistical analysis

Statistical analyses were performed using the software SigmaPlot 12.5 (Systat Software, Inc. USA). The Shapiro-Wilk test was used to assess the data for normality. Then, the t-test was used to test the expression levels of target genes between the Zhungeer and WL319HQ varieties. Results were considered significant at 5%.

## Additional files

Additional file 1: **Table S1.** DEGs generated from two varieties of alfalfa leaves. (DOCX 144 kb)

Additional file 2: **Table S2.** GO functional annotations and the number of DEGs statistics. (DOCX 18 kb)

Additional file 3: **Table S3.** KEGG pathway annotation of DEGs between two varieties of alfalfa leaves. (DOCX 26 kb)

Additional file 4: **Table S4.** Genes for RT-qPCR analysis and the list of GO ID. (DOCX 14 kb)

Additional file 5: **Table S5.** Primers used for RT-qPCR analysis. (DOCX 12 kb)

## Abbreviations

ABA: Abscisic acid
*ARF*: Auxin response factor
AuxREs: Auxin-responsive promoter elements
BLAST: Basic Local Alignment Search Tool
*CRY*: Cryptochrome
CTK: Cytokinin
*CTR1*: Constitutive triple response 1
DEGs: Differentially expressed genes
ETH: Ethylene
*ETR*: Ethylene receptor protein
GA: Gibberellin
*GH*: Indole-3-acetic acid-amido synthetase
GO: Gene Ontology
IAA: Indoleacetic acid
KEGG: Kyoto Encyclopedia of Genes and Genomes
KOG: Clusters of Orthologous Groups of proteins
*NCED3*: 9-cis-epoxycarotenoid dioxygenase 3
Nr: NCBI nonredundant protein
nt: Nucleotides
*PHYB*: Phytochrome B
*PIF3*: Phytochrome interacting factor 3
RT-qPCR: Real-time quantitative PCR
Swiss-Prot: A manually annotated and reviewed protein sequence database
*TUBA*: Tubulin alpha
