# Supplementary material for: Study on differentially expressed genes related to defoliation traits in two alfalfa varieties based on RNA-Seq
Source: BMC Genomics. 2018 Nov 7;19:807. doi: 10.1186/s12864-018-5180-1 (PMC6223052; doi:10.1186/s12864-018-5180-1)
Supplement: Supplementary file 1 — Table S1. DEGs generated from two varieties of alfalfa leaves. (DOCX 144 kb) [file 12864_2018_5180_MOESM1_ESM.docx]

| **GeneID** | **log2 Ratio**  **(WL319HQ/zhungeer)** | **FDR** | **Annotation** | |
| --- | --- | --- | --- | --- |
| Unigene0000195 | 11.53 | 0.033131925 | PREDICTED: DNA topoisomerase 2-binding protein 1-A isoform X4 [Vigna radiata var. radiata] [Vigna radiata] | |
| Unigene0000340 | 10.95 | 0.044488151 | 26S proteasome regulatory complex, ATPase RPT5 [Guillardia theta CCMP2712] | |
| Unigene0000518 | 12.60 | 0.00882385 | -- | |
| Unigene0000708 | 5.49 | 0.044261579 | -- | |
| Unigene0000719 | 3.57 | 0.037423884 | heat shock protein [Medicago sativa] | |
| Unigene0000991 | 12.65 | 0.004487042 | polysaccharide deacetylase [Klebsormidium flaccidum] | |
| Unigene0001031 | 3.03 | 0.000104005 | calcyclin-binding protein [Medicago truncatula] | |
| Unigene0001126 | 2.06 | 0.021118475 | disease resistance protein (TIR-NBS-LRR class) [Medicago truncatula] | |
| Unigene0001211 | 10.97 | 0.03016159 | PREDICTED: LOW QUALITY PROTEIN: vacuolar protein sorting-associated protein 21-like [Pyrus x bretschneideri] | |
| Unigene0001260 | 4.17 | 0.002062315 | PREDICTED: tubby-like F-box protein 8 [Cicer arietinum] | |
| Unigene0001584 | 5.84 | 0.007249236 | stress-induced receptor-like kinase [Medicago truncatula] | |
| Unigene0002127 | 11.73 | 0.009110915 | Leucine aminopeptidase 2, chloroplastic [Triticum urartu] | |
| Unigene0002157 | 12.45 | 0.008581051 | PLC-like phosphodiesterases superfamily protein [Theobroma cacao] | |
| Unigene0002207 | 4.21 | 0.009577835 | heat shock protein [Medicago sativa] | |
| Unigene0002226 | 16.04 | 0.001938094 | -- | |
| Unigene0002227 | 2.70 | 0.039623255 | DUF971 family protein [Medicago truncatula] | |
| Unigene0002298 | 12.09 | 0.002568075 | -- | |
| Unigene0002416 | 1.48 | 0.048800309 | RNA recognition motif [Medicago truncatula] | |
| Unigene0002483 | 5.35 | 0.040869057 | PREDICTED: protein NRDE2 homolog isoform X3 [Cicer arietinum] | |
| Unigene0002723 | 3.06 | 0.001198306 | PREDICTED: pyridoxal 5'-phosphate synthase-like subunit PDX1.2 [Cicer arietinum] | |
| Unigene0002827 | 2.89 | 0.014494493 | Os10g0192300 [Oryza sativa Japonica Group] | |
| Unigene0003059 | 2.23 | 0.003126452 | casein lytic proteinase B3 [Medicago truncatula] | |
| Unigene0003132 | 6.27 | 0.000434688 | ribosomal RNA small subunit methyltransferase G [Medicago truncatula] | |
| Unigene0003171 | 7.95 | 0.014691078 | -- | |
| Unigene0003236 | 3.41 | 0.000619904 | 17.6 kDa class I heat shock protein [Medicago truncatula] | |
| Unigene0003405 | 2.05 | 0.025394373 | multidrug resistance protein ABC transporter family protein [Medicago truncatula] | |
| Unigene0003586 | 4.77 | 0.00882385 | Polynucleotidyl transferase, Ribonuclease H fold [Medicago truncatula] | |
| Unigene0004126 | 1.97 | 0.028798599 | disease resistance protein (TIR-NBS-LRR class) [Medicago truncatula] | |
| Unigene0004130 | 8.64 | 0.013251228 | disease resistance protein (TIR-NBS-LRR class) [Medicago truncatula] | |
| Unigene0004203 | 14.31 | 0.041837285 | disease resistance protein (TIR-NBS-LRR class) [Medicago truncatula] | |
| Unigene0004216 | 1.68 | 0.045921376 | disease resistance protein (TIR-NBS-LRR class) [Medicago truncatula] | |
| Unigene0004270 | 2.66 | 0.041837285 | -- | |
| Unigene0004395 | 2.77 | 0.007448122 | Sec14p-like phosphatidylinositol transfer family protein [Medicago truncatula] | |
| Unigene0004423 | 4.14 | 0.005899902 | disease resistance protein (TIR-NBS-LRR class) [Medicago truncatula] | |
| Unigene0004434 | 2.71 | 0.033666906 | toll interleukin receptor [Phaseolus vulgaris] | |
| Unigene0004455 | 3.95 | 0.047893631 | disease resistance protein (TIR-NBS-LRR class) [Medicago truncatula] | |
| Unigene0004481 | 2.30 | 0.028148118 | disease resistance protein (TIR-NBS-LRR class) [Medicago truncatula] | |
| Unigene0004563 | 4.91 | 0.008907858 | hypothetical protein MtrDRAFT_AC150777g4v1 [Medicago truncatula] | |
| Unigene0004830 | 3.24 | 0.036498098 | F-box protein interaction domain protein [Medicago truncatula] | |
| Unigene0004937 | 2.31 | 0.030139298 | heat shock protein 15.9 [Medicago sativa] | |
| Unigene0005121 | 3.52 | 0.027623578 | peptidyl-prolyl cis-trans isomerase FKBP62-like protein [Medicago truncatula] | |
| Unigene0005442 | 5.09 | 0.006328811 | BAG domain protein [Medicago truncatula] | |
| Unigene0005443 | 3.19 | 0.013324709 | BAG domain protein [Medicago truncatula] | |
| Unigene0005445 | 4.51 | 0.000107618 | BAG domain protein [Medicago truncatula] | |
| Unigene0005522 | 3.88 | 1.97E-05 | wound-responsive family protein [Medicago truncatula] | |
| Unigene0005618 | 6.64 | 0.017995618 | zinc-ribbon domain protein [Medicago truncatula] | |
| Unigene0005718 | 11.17 | 0.032968356 | PREDICTED: plasma membrane ATPase-like [Oryza brachyantha] | |
| Unigene0005913 | 1.82 | 0.011999868 | activator of 90 kDa heat shock ATPase-like protein [Medicago truncatula] | |
| Unigene0005981 | 3.05 | 0.044590622 | -- | |
| Unigene0006280 | 3.46 | 0.010477551 | ARF guanine-nucleotide exchange factor GNOM protein [Medicago truncatula] | |
| Unigene0006639 | 10.16 | 0.03484521 | -- | |
| Unigene0007140 | 13.21 | 0.037978533 | -- | |
| Unigene0007182 | 11.46 | 0.006963778 | transmembrane protein, putative [Medicago truncatula] | |
| Unigene0007185 | 2.66 | 0.032023979 | P-loop nucleoside triphosphate hydrolase superfamily protein [Medicago truncatula] | |
| Unigene0007220 | 3.65 | 0.031044732 | PREDICTED: extensin-1-like [Cicer arietinum] | |
| Unigene0007665 | 2.75 | 2.61E-05 | TPR1 [Medicago sativa] | |
| Unigene0007673 | 2.39 | 0.011081909 | ribosomal protein S8 [Medicago truncatula] | |
| Unigene0007783 | 10.55 | 0.02787007 | 3-hydroxy-3-methylglutaryl-coenzyme A reductase-like protein [Medicago truncatula] | |
| Unigene0008075 | 2.36 | 0.006774791 | armadillo/beta-catenin-like repeat protein [Medicago truncatula] | |
| Unigene0008091 | 1.95 | 0.040552966 | ribulose bisphosphate carboxylase/oxygenase activase [Medicago truncatula] | |
| Unigene0008286 | 1.93 | 0.010092351 | seed specific protein Bn15D17A [Medicago truncatula] | |
| Unigene0008400 | 11.54 | 0.020819723 | PREDICTED: probable voltage-gated potassium channel subunit beta [Daucus carota subsp. sativus] [Daucus carota] | |
| Unigene0008605 | 2.52 | 0.006521489 | localized small heat shock protein [Medicago truncatula] | |
| Unigene0008899 | 12.35 | 0.006774791 | -- | |
| Unigene0009216 | 2.54 | 0.006927506 | PREDICTED: TMV resistance protein N-like [Cicer arietinum] | |
| Unigene0009414 | 12.23 | 0.01256078 | -- | |
| Unigene0009465 | 10.85 | 0.039623255 | succinyl-CoA synthetase beta subunit [Galdieria sulphuraria] | |
| Unigene0010933 | 12.44 | 0.001499929 | -- | |
| Unigene0011374 | 12.19 | 0.00395914 | -- | |
| Unigene0011585 | 8.09 | 0.015828635 | -- | |
| Unigene0011752 | 3.87 | 0.049193847 | Gag-pol polyprotein-like protein [Theobroma cacao] | |
| Unigene0011847 | 1.88 | 0.026211841 | multiprotein bridging factor 1b-like protein [Medicago truncatula] | |
| Unigene0012097 | 2.86 | 0.039623255 | BAG domain protein [Medicago truncatula] | |
| Unigene0012214 | 2.70 | 0.010222982 | universal stress family protein [Medicago truncatula] | |
| Unigene0012341 | 7.74 | 4.96E-06 | 22.0 kDa class IV heat shock protein [Medicago truncatula] | |
| Unigene0012476 | 11.07 | 0.041414718 | PREDICTED: proliferating cell nuclear antigen large form [Daucus carota subsp. sativus] [Daucus carota] | |
| Unigene0012528 | 2.76 | 0.000345569 | wound-responsive family protein [Medicago truncatula] | |
| Unigene0012611 | 13.16 | 0.005852769 | 60S ribosomal protein L29e [Galdieria sulphuraria] | |
| Unigene0012721 | 11.11 | 0.026382019 | PREDICTED: mannose-1-phosphate guanylyltransferase 1-like [Solanum lycopersicum] | |
| Unigene0013215 | 10.85 | 0.028420111 | -- | |
| Unigene0013221 | 15.39 | 0.005044898 | -- | |
| Unigene0013297 | 1.67 | 0.025689576 | endonuclease/exonuclease/phosphatase family protein [Medicago truncatula] | |
| Unigene0013300 | 10.87 | 0.034614998 | -- | |
| Unigene0013454 | 10.81 | 0.048402456 | PREDICTED: DEAD-box ATP-dependent RNA helicase 34 [Oryza sativa Japonica Group] | |
| Unigene0013526 | 9.72 | 0.010455751 | unknown [Populus trichocarpa x Populus deltoides] | |
| Unigene0013575 | 6.33 | 0.02812805 | -- | |
| Unigene0013581 | 3.11 | 0.017767289 | heat shock protein 17.6 [Medicago sativa] | |
| Unigene0013622 | 10.92 | 0.047893631 | PREDICTED: chromo domain-containing protein LHP1-like isoform X1 [Glycine max] | |
| Unigene0013643 | 7.87 | 0.029576515 | Retrotransposable element Tf2, partial [Cajanus cajan] | |
| Unigene0013660 | 11.91 | 0.031284209 | Cytochrome c oxidase subunit 6B [Aegilops tauschii] | |
| Unigene0013690 | 12.81 | 0.018846081 | large subunit ribosomal protein L37Ae, cytoplasmic [Guillardia theta CCMP2712] | |
| Unigene0013698 | 8.14 | 0.030270637 | PREDICTED: serine carboxypeptidase-like 35 [Elaeis guineensis] | |
| Unigene0013713 | 5.14 | 0.013194965 | importin subunit alpha [Medicago truncatula] | |
| Unigene0013778 | 4.69 | 0.034700686 | -- | |
| Unigene0013813 | 11.40 | 0.006927506 | Na+-ATPase [Chondrus crispus] | |
| Unigene0013840 | 11.74 | 0.018024909 | PREDICTED: RNA-binding protein Nova-1-like isoform X2 [Elaeis guineensis] | |
| Unigene0013888 | 13.22 | 0.005852769 | Ribosomal protein S5/S7, eukaryotic/archaeal [Ostreococcus tauri] | |
| Unigene0013895 | 11.65 | 0.021256326 | PREDICTED: autophagy-related protein 8d-like [Camelina sativa] | |
| Unigene0013908 | 11.00 | 0.015278708 | -- | |
| Unigene0013911 | 9.47 | 0.018975927 | PREDICTED: calnexin homolog 1-like [Nicotiana tabacum] | |
| Unigene0013913 | 11.69 | 0.015671833 | BnaC01g17280D [Brassica napus] | |
| Unigene0013914 | 7.98 | 0.040299854 | PREDICTED: reticuline oxidase-like protein [Gossypium hirsutum] | |
| Unigene0013920 | 10.93 | 0.039623255 | PREDICTED: 2-isopropylmalate synthase 1, chloroplastic-like [Vigna radiata var. radiata] [Vigna radiata] | |
| Unigene0013924 | 8.84 | 0.018550717 | pyruvate carboxylase [Blastocystis sp. subtype 4] | |
| Unigene0013934 | 10.45 | 0.000316202 | PREDICTED: heat shock 70 kDa protein-like [Arachis duranensis] | |
| Unigene0013936 | 11.24 | 0.047555749 | hypothetical protein ZOSMA_198G00050 [Zostera marina] | |
| Unigene0013943 | 13.44 | 6.44E-05 | expressed protein [Oryza sativa Japonica Group] | |
| Unigene0013953 | 11.18 | 0.01946615 | PREDICTED: protein argonaute 5 [Tarenaya hassleriana] | |
| Unigene0013979 | 12.66 | 0.007413329 | ADP-ribosylation factor [Zostera marina] | |
| Unigene0013980 | 10.40 | 0.044761345 | NOSIC [Ostreococcus tauri] | |
| Unigene0014010 | 2.93 | 0.008236288 | Retrovirus-related Pol polyprotein from transposon TNT 1-94 [Cajanus cajan] | |
| Unigene0014069 | 13.13 | 0.009914905 | -- | |
| Unigene0014081 | 13.88 | 0.005542093 | histone 3 [Conocephalum conicum] | |
| Unigene0014089 | 11.56 | 0.037978533 | succinate dehydrogenase iron sulfur protein [Phaeodactylum tricornutum CCAP 1055/1] | |
| Unigene0014098 | 12.84 | 0.00415412 | PREDICTED: pathogenesis-related protein 5-like [Camelina sativa] | |
| Unigene0014105 | 10.82 | 0.041837285 | transcription factor IIA, alpha/beta subunit [Micromonas pusilla CCMP1545] | |
| Unigene0014108 | 8.85 | 0.006917034 | -- | |
| Unigene0014126 | 3.13 | 0.003440308 | hypothetical protein MTR_1155s0010 [Medicago truncatula] | |
| Unigene0014206 | 11.11 | 0.031587979 | cytoplasmic DExD/H-box RNA helicase [Coccomyxa subellipsoidea C-169] | |
| Unigene0014406 | 12.15 | 0.02787007 | hypothetical protein KFL_001190120 [Klebsormidium flaccidum] | |
| Unigene0014544 | 13.14 | 0.012960417 | PREDICTED: Werner syndrome ATP-dependent helicase [Vigna angularis] | |
| Unigene0014561 | 3.40 | 0.025249306 | LRR/extensin 2 [Medicago truncatula] | |
| Unigene0014592 | 10.94 | 0.036646766 | PREDICTED: organic cation/carnitine transporter 7-like [Erythranthe guttata] | |
| Unigene0014595 | 5.32 | 0.016598092 | -- | |
| Unigene0014607 | 12.96 | 0.011089935 | -- | |
| Unigene0014658 | 3.02 | 0.000572541 | casein lytic proteinase B3 [Medicago truncatula] | |
| Unigene0014908 | 3.04 | 0.048624329 | LURP-one-like protein [Medicago truncatula] | |
| Unigene0014915 | 10.83 | 0.022591424 | -- | |
| Unigene0015010 | 11.63 | 0.032968356 | PREDICTED: probable 6-phosphogluconolactonase 4, chloroplastic [Arachis ipaensis] | |
| Unigene0015174 | 13.01 | 0.00788769 | superoxide dismutase (mitochondrion) [Triticum aestivum] | |
| Unigene0015207 | 14.03 | 0.000714691 | ribosomal protein S3Ae [Coccomyxa subellipsoidea C-169] | |
| Unigene0015212 | 10.81 | 0.00882385 | PREDICTED: heat shock 70 kDa protein-like [Arachis duranensis] | |
| Unigene0015251 | 10.56 | 0.044449893 | -- | |
| Unigene0015409 | 12.71 | 0.006774791 | cold-inducible rna-binding protein [Nannochloropsis gaditana] | |
| Unigene0015415 | 8.29 | 0.040692042 | hypothetical protein VITISV_041860, partial [Vitis vinifera] | |
| Unigene0015425 | 11.43 | 0.01596238 | PREDICTED: probable serine/threonine-protein kinase DDB_G0291350 [Elaeis guineensis] | |
| Unigene0015427 | 12.04 | 0.030409802 | -- | |
| Unigene0015456 | 12.65 | 0.016598092 | -- | |
| Unigene0015656 | 8.08 | 0.040655182 | -- | |
| Unigene0015757 | 8.34 | 0.037494188 | PREDICTED: 60S ribosomal protein L4 [Vigna radiata var. radiata] [Vigna radiata] | |
| Unigene0015932 | 11.94 | 0.046068717 | -- | |
| Unigene0016088 | 11.40 | 0.040299854 | -- | |
| Unigene0016103 | 11.02 | 0.03016159 | -- | |
| Unigene0016278 | 12.80 | 0.019133271 | PREDICTED: 60S ribosomal protein L9 [Nelumbo nucifera] | |
| Unigene0016291 | 13.01 | 0.00788769 | translation initiation factor eIF-5A [Arabidopsis thaliana] | |
| Unigene0016333 | 11.07 | 0.013332943 | ABC transporter, ATP-binding protein, partial [Galdieria sulphuraria] | |
| Unigene0016348 | 11.52 | 0.010631918 | eukaryotic translation initiation factor 2c [Medicago truncatula] | |
| Unigene0016378 | 10.94 | 0.016824954 | 22.0 kDa class IV heat shock protein [Medicago truncatula] | |
| Unigene0016408 | 9.99 | 0.03484521 | -- | |
| Unigene0016446 | 12.83 | 0.010084406 | ATP synthase subunit gamma, mitochondrial [Auxenochlorella protothecoides] | |
| Unigene0016507 | 11.54 | 0.04954846 | -- | |
| Unigene0016844 | 11.12 | 0.034614998 | -- | |
| Unigene0016898 | 13.17 | 0.00098938 | alpha,alpha-trehalose-phosphate synthase (UDP-forming) [Nannochloropsis gaditana CCMP526] | |
| Unigene0016994 | 11.54 | 0.00820457 | atp-citrate synthase [Phaeodactylum tricornutum CCAP 1055/1] | |
| Unigene0017167 | 11.24 | 0.02660871 | PREDICTED: katanin p80 WD40 repeat-containing subunit B1 homolog isoform X2 [Cucumis sativus] | |
| Unigene0017189 | 10.93 | 0.018501362 | PREDICTED: sugar carrier protein C-like [Oryza brachyantha] | |
| Unigene0017308 | 9.66 | 0.011703785 | 60S ribosomal protein L7Ae isoform 2 [Galdieria sulphuraria] | |
| Unigene0017310 | 13.76 | 0.00677181 | Hsp10 [Crypthecodinium cohnii] | |
| Unigene0017511 | 11.89 | 0.009186533 | PREDICTED: pyruvate dehydrogenase E1 component subunit beta-1, mitochondrial-like [Camelina sativa] | |
| Unigene0017606 | 11.45 | 0.014698166 | aldehyde dehydrogenase [Coccomyxa subellipsoidea C-169] | |
| Unigene0017668 | 10.79 | 0.029436128 | -- | |
| Unigene0017716 | 12.79 | 0.015548461 | PREDICTED: ATP synthase subunit delta', mitochondrial [Erythranthe guttata] | |
| Unigene0017760 | 10.87 | 0.022550039 | -- | |
| Unigene0018133 | 12.19 | 0.018687328 | -- | |
| Unigene0018357 | 10.69 | 0.016680432 | phosphoenolpyruvate carboxykinase, partial [Urochloa villosa] | |
| Unigene0018588 | 11.95 | 0.010993135 | -- | |
| Unigene0019035 | 10.79 | 0.040791389 | PREDICTED: apoptosis-inducing factor homolog B-like isoform X1 [Daucus carota subsp. sativus] [Daucus carota] | |
| Unigene0019037 | 13.23 | 0.00677181 | -- | |
| Unigene0019181 | 12.94 | 0.02660871 | ribosomal protein L31 [Cyanophora paradoxa] | |
| Unigene0019322 | 10.59 | 0.040791389 | -- | |
| Unigene0019368 | 9.78 | 0.009686075 | polyubiquitin protein, partial [Helianthus annuus] | |
| Unigene0019369 | 9.02 | 0.041414718 | polyubiquitin, partial [Cicer arietinum] | |
| Unigene0019426 | 13.03 | 0.002174916 | unknown [Zea mays] | |
| Unigene0019496 | 9.29 | 0.005549865 | 60S ribosomal protein L13a-2 [Zea mays] | |
| Unigene0019562 | 12.38 | 0.011409804 | Protein HEADING DATE 3B [Auxenochlorella protothecoides] | |
| Unigene0019735 | 9.17 | 0.025689576 | full-length thiazole biosynthetic enzyme [Chlamydomonas reinhardtii] | |
| Unigene0019871 | 11.49 | 0.037605515 | chloroplast RNA binding protein [Mesembryanthemum crystallinum] | |
| Unigene0019941 | 12.64 | 0.028865076 | multiprotein bridging factor [Micromonas commoda] | |
| Unigene0020015 | 11.39 | 0.046824822 | BnaA04g03470D [Brassica napus] | |
| Unigene0020083 | 11.19 | 0.037605515 | -- | |
| Unigene0020176 | 4.39 | 1.65E-05 | 17.6 kDa class I heat shock protein [Medicago truncatula] | |
| Unigene0020226 | 10.76 | 0.035073564 | Serine/threonine protein phosphatase 7 long form isogeny, partial [Cajanus cajan] | |
| Unigene0020252 | 13.51 | 0.003914265 | -- | |
| Unigene0020317 | 12.31 | 0.014353925 | inorganic pyrophosphatase [Galdieria sulphuraria] | |
| Unigene0020382 | 13.15 | 0.006189804 | PREDICTED: 60S ribosomal protein L3 [Eucalyptus grandis] | |
| Unigene0020515 | 11.47 | 0.007448122 | Isopropylmalate dehydratase [Guillardia theta CCMP2712] | |
| Unigene0020518 | 8.62 | 0.026300772 | Mitochondrial outer membrane protein porin 1 [Auxenochlorella protothecoides] | |
| Unigene0020628 | 11.85 | 0.006916976 | transketolase [Phaeodactylum tricornutum CCAP 1055/1] | |
| Unigene0020696 | 11.22 | 0.030978859 | saccharopine dehydrogenase [Coccomyxa subellipsoidea C-169] | |
| Unigene0020706 | 12.57 | 0.008581051 | mitochondrial phosphate carrier 1 [Volvox carteri f. nagariensis] | |
| Unigene0020761 | 10.82 | 0.00055554 | -- | |
| Unigene0020927 | 12.62 | 0.030463833 | 40S ribosomal protein S28 [Triticum urartu] | |
| Unigene0021009 | 10.80 | 0.031599821 | abc transporter [Klebsormidium flaccidum] | |
| Unigene0021059 | 9.11 | 0.023959119 | 60S ribosomal protein L19-2 [Arabidopsis thaliana] | |
| Unigene0021118 | 6.85 | 0.016315959 | drug resistance transporter-like ABC domain protein [Medicago truncatula] | |
| Unigene0021154 | 11.21 | 0.04121937 | PREDICTED: proteasome subunit alpha type-6-A-like [Camelina sativa] | |
| Unigene0021189 | 12.82 | 0.031401746 | 60S ribosomal protein L27e [Galdieria sulphuraria] | |
| Unigene0021210 | 6.37 | 0.049046268 | -- | |
| Unigene0021261 | 11.50 | 0.026746844 | -- | |
| Unigene0021293 | 12.93 | 0.020809829 | cytoplasmic ribosomal protein S13 [Panax ginseng] | |
| Unigene0021360 | 13.12 | 0.013111564 | PREDICTED: 60S ribosomal protein L35-2-like [Tarenaya hassleriana] | |
| Unigene0021385 | 10.74 | 0.024735161 | TCP-1/cpn60 chaperonin family protein [Klebsormidium flaccidum] | |
| Unigene0021511 | 11.53 | 0.046638498 | PREDICTED: small nuclear ribonucleoprotein-associated protein B [Gossypium raimondii] | |
| Unigene0021599 | 8.16 | 0.037223895 | PREDICTED: probable calcium-binding protein CML49 [Citrus sinensis] | |
| Unigene0021639 | 14.84 | 0.001127994 | DNAJ heat shock family protein [Dorcoceras hygrometricum] | |
| Unigene0021646 | 5.62 | 0.040412303 | hypothetical protein TSUD_90240 [Trifolium subterraneum] | |
| Unigene0021657 | 4.98 | 0.022478593 | wound-responsive family protein [Medicago truncatula] | |
| Unigene0021726 | 11.09 | 0.03224367 | -- | |
| Unigene0021778 | 13.00 | 0.00936597 | PREDICTED: protein OPI10 homolog [Solanum tuberosum] | |
| Unigene0021819 | 10.89 | 0.021798922 | -- | |
| Unigene0021925 | 12.52 | 0.025689576 | hypothetical protein SELMODRAFT_443235, partial [Selaginella moellendorffii] | |
| Unigene0021935 | 13.45 | 0.011409804 | -- | |
| Unigene0022416 | 11.96 | 0.013991275 | -- | |
| Unigene0022457 | 11.39 | 0.041837285 | ABC transporter [Klebsormidium flaccidum] | |
| Unigene0022458 | 11.47 | 0.028740008 | elongation factor EF-3 [Coccomyxa subellipsoidea C-169] | |
| Unigene0022527 | 10.90 | 0.026040172 | -- | |
| Unigene0022547 | 3.65 | 0.026211841 | -- | |
| Unigene0022595 | 12.95 | 0.004231481 | MnSOD, partial [Hevea brasiliensis] | |
| Unigene0022757 | 12.89 | 0.00882385 | -- | |
| Unigene0022781 | 12.40 | 0.02216115 | -- | |
| Unigene0022803 | 10.74 | 0.002589195 | -- | |
| Unigene0022807 | 12.10 | 0.021118475 | FKBP-type peptidyl-prolyl cis-trans isomerase Fkh1 [Blastocystis sp. NandII] | |
| Unigene0022832 | 10.82 | 0.042398666 | Glycine-rich RNA-binding protein 4, mitochondrial [Zostera marina] | |
| Unigene0022889 | 10.75 | 0.014854974 | E3 ubiquitin-protein ligase UPL2 [Morus notabilis] | |
| Unigene0022913 | 11.50 | 0.038459428 | binding partner of acd11 1 [Arabidopsis thaliana] | |
| Unigene0022914 | 12.26 | 0.008426964 | lysosomal aspartic protease-like protein [Chrysochromulina sp. CCMP291] | |
| Unigene0022946 | 8.75 | 0.042661507 | probable 1,4-benzoquinone reductase [Cyanidioschyzon merolae strain 10D] [Cyanidioschyzon merolae] | |
| Unigene0022947 | 12.13 | 0.013991275 | alcohol dehydrogenase (NADP+) [Klebsormidium flaccidum] | |
| Unigene0023040 | 10.69 | 0.004219021 | PREDICTED: chaperone protein ClpB2, chloroplastic-like [Zea mays] | |
| Unigene0023231 | 11.52 | 0.01570027 | hypothetical protein JH06_4311 [Blastocystis sp. subtype 4] | |
| Unigene0023281 | 11.94 | 0.018169753 | small rab-related GTPase [Chlamydomonas reinhardtii] | |
| Unigene0023363 | 11.90 | 0.006774791 | elongation factor EF-2 [Galdieria sulphuraria] | |
| Unigene0023402 | 8.43 | 0.031401746 | mitochondrial processing peptidase [Klebsormidium flaccidum] | |
| Unigene0023416 | 11.32 | 0.013324709 | cytosolic glucose-6-phosphate isomerase [Pavlova lutheri] | |
| Unigene0023474 | 12.08 | 0.018687328 | translation initiation factor SU [Coccomyxa subellipsoidea C-169] | |
| Unigene0023515 | 4.02 | 0.049496927 | mediator of RNA polymerase II transcription subunit 16 [Medicago truncatula] | |
| Unigene0023526 | 12.67 | 0.008236288 | unknown [Populus trichocarpa x Populus deltoides] | |
| Unigene0023527 | 12.23 | 0.007815975 | nucleosome/chromatin assembly factor group protein [Medicago truncatula] | |
| Unigene0023541 | 11.62 | 0.014854974 | omega-6 fatty acid desaturase delta-12 [Nannochloropsis gaditana CCMP526] | |
| Unigene0023776 | 12.93 | 0.010993135 | elongation factor EF-1 beta subunit [Galdieria sulphuraria] | |
| Unigene0023836 | 13.36 | 0.003519832 | actin [Rhodomonas sp. CCMP768] | |
| Unigene0023889 | 9.01 | 0.029714663 | vitamine B12-independent methionine synthase [Cyanidioschyzon merolae strain 10D] [Cyanidioschyzon merolae] | |
| Unigene0023906 | 4.05 | 0.028420111 | heat shock protein 81-2 [Medicago truncatula] | |
| Unigene0023919 | 5.93 | 0.042439732 | glycoside hydrolase family 81 protein [Medicago truncatula] | |
| Unigene0023950 | 11.38 | 0.035526624 | store-operated calcium entry-associated regulatory factor precursor [Blastocystis sp. NandII] | |
| Unigene0023989 | 9.84 | 0.000681065 | PREDICTED: heat shock protein 83 [Phoenix dactylifera] | |
| Unigene0024038 | 8.97 | 0.019980397 | beta-1 tubulin [Dendrobium catenatum] | |
| Unigene0024048 | 11.05 | 0.034661967 | GTP-binding protein [Klebsormidium flaccidum] | |
| Unigene0024140 | 11.04 | 0.034028894 | -- | |
| Unigene0024200 | 12.78 | 0.012347801 | -- | |
| Unigene0024221 | 12.13 | 0.032711496 | V-type proton ATPase subunit c5 [Ananas comosus] | |
| Unigene0024248 | 5.55 | 0.032968356 | 1-phosphatidylinositol phosphodiesterase-like protein [Medicago truncatula] | |
| Unigene0024282 | 9.93 | 0.04954846 | Polynucleotidyl transferase, Ribonuclease H fold [Medicago truncatula] | |
| Unigene0024318 | 13.34 | 0.008510306 | 40s ribosomal protein [Chrysochromulina sp. CCMP291] | |
| Unigene0024319 | 12.86 | 0.012172516 | ribosomal protein S7, component of cytosolic 80S ribosome and 40S small subunit [Chlamydomonas reinhardtii] | |
| Unigene0024355 | 10.86 | 0.02164242 | UBX domain-containing protein 1 [Klebsormidium flaccidum] | |
| Unigene0024478 | 10.58 | 0.046611805 | MFS transporter, SP family, sugar:H+ symporter [Galdieria sulphuraria] | |
| Unigene0024493 | 13.15 | 0.006328811 | -- | |
| Unigene0024497 | 11.64 | 0.031928066 | Histone H4 [Zostera marina] | |
| Unigene0024498 | 13.29 | 0.042782194 | Histone H4 [Zostera marina] | |
| Unigene0024533 | 11.03 | 0.035187016 | PREDICTED: trans-resveratrol di-O-methyltransferase-like [Sesamum indicum] | |
| Unigene0024639 | 12.32 | 0.046638498 | Histone H4 [Zostera marina] | |
| Unigene0024713 | 7.94 | 0.007448122 | PREDICTED: protein TAR1-like, partial [Eucalyptus grandis] | |
| Unigene0024740 | 8.95 | 0.019563255 | -- | |
| Unigene0024790 | 12.70 | 0.004263413 | trehalose-6-phosphate synthase [Pyropia haitanensis] | |
| Unigene0024793 | 11.24 | 0.041156046 | lipase family protein [Nannochloropsis gaditana] | |
| Unigene0024845 | 7.91 | 0.03016159 | elongation factor 1-gamma [Blastocystis sp. NandII] | |
| Unigene0024989 | 9.17 | 0.026908499 | -- | |
| Unigene0025032 | 12.70 | 0.006407309 | Tubulin alpha chain [Morus notabilis] | |
| Unigene0025059 | 12.39 | 0.00677181 | carbamoyl-phosphate synthase small subunit [Blastocystis sp. NandII] | |
| Unigene0025216 | 9.53 | 0.008395505 | -- | |
| Unigene0025296 | 13.20 | 0.002735544 | adenosylhomocysteinase [Galdieria sulphuraria] | |
| Unigene0025382 | 11.78 | 0.009981876 | phosphatase isoform 1 [Galdieria sulphuraria] | |
| Unigene0025408 | 11.25 | 0.032449834 | arginine/serine-rich splicing factor 6 [Ectocarpus siliculosus] | |
| Unigene0025479 | 10.46 | 0.002995641 | heat shock protein [Galdieria sulphuraria] | |
| Unigene0025481 | 10.75 | 0.007249236 | heat shock protein [Galdieria sulphuraria] | |
| Unigene0025512 | 9.67 | 0.003875774 | -- | |
| Unigene0025546 | 7.96 | 0.041363271 | PREDICTED: peroxiredoxin-2B [Capsicum annuum] | |
| Unigene0025560 | 10.33 | 0.015548461 | -- | |
| Unigene0025684 | 9.39 | 0.016460947 | heat shock protein 70-1 [Gracilariopsis lemaneiformis] | |
| Unigene0025693 | 11.38 | 0.012347801 | -- | |
| Unigene0025755 | 12.34 | 0.017184914 | PREDICTED: THO complex subunit 4A-like [Prunus mume] | |
| Unigene0025809 | 14.97 | 0.002974456 | -- | |
| Unigene0025866 | 7.19 | 7.45E-12 | 17.6 kDa class I heat shock protein [Medicago truncatula] | |
| Unigene0025870 | 11.95 | 0.01256078 | PREDICTED: pyridoxal biosynthesis protein PDX1.3 [Eucalyptus grandis] | |
| Unigene0025992 | 13.05 | 0.000785746 | PREDICTED: ATP synthase subunit alpha, mitochondrial [Solanum lycopersicum] | |
| Unigene0026023 | 10.56 | 0.037078211 | PREDICTED: cyclase-associated protein 1 [Oryza brachyantha] | |
| Unigene0026432 | 9.63 | 0.006467823 | heat shock protein [Galdieria sulphuraria] | |
| Unigene0026441 | 13.14 | 0.016598092 | PREDICTED: 40S ribosomal protein S19-2 isoform X1 [Amborella trichopoda] | |
| Unigene0026513 | 12.57 | 0.037423884 | -- | |
| Unigene0026530 | 13.34 | 0.011081909 | 40s ribosomal protein s8 [Nannochloropsis gaditana] | |
| Unigene0026593 | 8.57 | 0.029436128 | periaxin-like protein [Arabidopsis thaliana] | |
| Unigene0026602 | 6.30 | 0.021543896 | wound-responsive family protein [Medicago truncatula] | |
| Unigene0026610 | 11.37 | 0.028805397 | -- | |
| Unigene0026622 | 13.51 | 0.006834371 | -- | |
| Unigene0026801 | 11.94 | 0.01256078 | -- | |
| Unigene0026874 | 4.68 | 0.029961991 | DUF4378 domain protein [Medicago truncatula] | |
| Unigene0026945 | 8.92 | 0.018016132 | exo-beta- -glucanase [Nannochloropsis gaditana] | |
| Unigene0027159 | 9.37 | 0.008979743 | PREDICTED: hsp70-Hsp90 organizing protein 3 [Ricinus communis] | |
| Unigene0027194 | 11.79 | 0.008143504 | -- | |
| Unigene0027251 | 11.43 | 0.017642327 | -- | |
| Unigene0027280 | 13.39 | 0.006033083 | -- | |
| Unigene0027344 | 9.34 | 0.002787102 | -- | |
| Unigene0027353 | 11.09 | 0.02660871 | S-Adenosyl-Methionine synthetase [Ectocarpus siliculosus] | |
| Unigene0027792 | 13.15 | 0.00882385 | gtp-binding nuclear protein ran [Nannochloropsis gaditana] | |
| Unigene0027794 | 8.88 | 0.015278708 | PREDICTED: malate dehydrogenase, mitochondrial [Nicotiana sylvestris] | |
| Unigene0027819 | 11.30 | 0.018016132 | PREDICTED: chaperone protein dnaJ GFA2, mitochondrial-like [Phoenix dactylifera] | |
| Unigene0027912 | 8.01 | 0.044948435 | -- | |
| Unigene0027913 | 11.73 | 0.027773665 | PREDICTED: cysteine and histidine-rich domain-containing protein RAR1 isoform X2 [Gossypium raimondii] | |
| Unigene0028010 | 13.61 | 0.00677181 | PREDICTED: 60S ribosomal protein L6-1-like [Brassica oleracea var. oleracea] [Brassica oleracea] | |
| Unigene0028025 | 2.01 | 0.015573279 | BnaAnng07940D [Brassica napus] | |
| Unigene0028055 | 11.28 | 0.008979743 | PREDICTED: peroxidase 2-like [Ziziphus jujuba] | |
| Unigene0028101 | 11.97 | 0.006328811 | Retrovirus-related Pol polyprotein from transposon 297 family [Cajanus cajan] | |
| Unigene0028145 | 12.24 | 0.012856933 | short-chain dehydrogenase/reductase SDR isoform 1 [Galdieria sulphuraria] | |
| Unigene0028229 | 11.54 | 0.008668785 | luminal-binding protein 5-like protein [Chrysochromulina sp. CCMP291] | |
| Unigene0028285 | 11.50 | 0.018253185 | PREDICTED: protein disulfide-isomerase-like [Daucus carota subsp. sativus] [Daucus carota] | |
| Unigene0028289 | 10.40 | 0.003816898 | -- | |
| Unigene0028306 | 3.60 | 0.011348498 | mRNA splicing factor, thioredoxin-like U5 snRNP protein [Medicago truncatula] | |
| Unigene0028341 | 5.52 | 0.001209088 | Transposon Ty3-G Gag-Pol polyprotein [Cajanus cajan] | |
| Unigene0028692 | 2.76 | 0.010607994 | zinc-ribbon domain protein [Medicago truncatula] | |
| Unigene0028732 | 8.41 | 0.027108535 | -- | |
| Unigene0028849 | 11.67 | 0.014171769 | TATA-box binding protein, component of TFIID and TFIIIB [Physcomitrella patens] | |
| Unigene0029025 | 11.18 | 0.032702027 | Translation elongation factor EF-Tu, mitochondrial [Chondrus crispus] | |
| Unigene0029062 | 8.35 | 0.033517882 | PREDICTED: ATPase 9, plasma membrane-type [Tarenaya hassleriana] | |
| Unigene0029366 | 12.68 | 0.017991899 | histone 3 [Conocephalum conicum] | |
| Unigene0029574 | 12.33 | 0.019822935 | -- | |
| Unigene0029631 | 5.42 | 0.000373891 | cytosolic class II small heat-shock protein [Medicago truncatula] | |
| Unigene0029635 | 9.40 | 0.017475756 | ser/thr kinase [Chlamydomonas reinhardtii] | |
| Unigene0029639 | 11.13 | 0.029781319 | glycoside hydrolase family 16 protein [Chrysochromulina sp. CCMP291] | |
| Unigene0029723 | 10.91 | 0.011929214 | PREDICTED: enoyl-CoA hydratase 2, peroxisomal [Solanum pennellii] | |
| Unigene0029919 | 3.34 | 0.000619904 | ethylene response factor [Medicago truncatula] | |
| Unigene0030023 | 2.38 | 0.006658661 | SAUR-like auxin-responsive family protein [Medicago truncatula] | |
| Unigene0030311 | 11.76 | 0.025210473 | thaumatin-like protein [Triticum aestivum] | |
| Unigene0030522 | 3.34 | 0.009025905 | BnaA10g23780D [Brassica napus] | |
| Unigene0030523 | 13.69 | 0.014854974 | -- | |
| Unigene0030548 | 11.71 | 0.025799838 | disease resistance protein (TIR-NBS-LRR class) [Medicago truncatula] | |
| Unigene0030777 | 3.81 | 0.025689576 | cytosolic class II small heat-shock protein [Medicago truncatula] | |
| Unigene0030884 | 8.56 | 0.048624329 | -- | |
| Unigene0030898 | 11.47 | 0.048126764 | -- | |
| Unigene0030931 | 8.87 | 0.03203935 | PREDICTED: dnaJ protein homolog 2 isoform X2 [Solanum pennellii] | |
| Unigene0030932 | 11.18 | 0.013348202 | hypothetical protein SELMODRAFT_408477 [Selaginella moellendorffii] | |
| Unigene0031083 | 1.91 | 0.047989811 | PREDICTED: LOW QUALITY PROTEIN: fructose-bisphosphate aldolase, cytoplasmic isozyme-like [Cicer arietinum] | |
| Unigene0031334 | 10.30 | 0.002890743 | mitochondrial chaperonin 60 precursor [Chlamydomonas reinhardtii] | |
| Unigene0031493 | 2.60 | 0.028539731 | EF hand calcium-binding family protein [Medicago truncatula] | |
| Unigene0031505 | 10.32 | 0.006624208 | -- | |
| Unigene0031524 | 5.61 | 0.000316202 | 17.6 kDa class I heat shock protein [Medicago truncatula] | |
| Unigene0031677 | 11.45 | 0.013921853 | -- | |
| Unigene0031700 | 5.80 | 0.000456676 | disease resistance protein (TIR-NBS-LRR class) [Medicago truncatula] | |
| Unigene0031913 | 11.44 | 0.006521489 | mitochondrial aconitate hydratase [Volvox carteri f. nagariensis] | |
| Unigene0032009 | 12.67 | 0.004231481 | subtilisin-related vacuolar protease [Emiliania huxleyi CCMP1516] | |
| Unigene0032368 | 2.81 | 0.033666906 | universal stress family protein [Medicago truncatula] | |
| Unigene0032382 | 4.46 | 0.043488797 | -- | |
| Unigene0032531 | 4.38 | 0.022516628 | galactinol synthase [Medicago truncatula] | |
| Unigene0032543 | 3.70 | 0.028836587 | serine carboxypeptidase-like protein [Medicago truncatula] | |
| Unigene0032699 | 4.09 | 0.011409804 | disease resistance protein (TIR-NBS-LRR class) [Medicago truncatula] | |
| Unigene0032887 | 3.81 | 0.049496927 | indole-3-acetic acid-amido synthetase [Medicago truncatula] | |
| Unigene0033385 | 8.99 | 0.021118475 | 40S ribosomal protein S6e [Galdieria sulphuraria] | |
| Unigene0033544 | 13.06 | 0.00882385 | 30S ribosomal protein S5 [Zostera marina] | |
| Unigene0033548 | 12.18 | 0.000400314 | -- | |
| Unigene0033871 | 13.92 | 0.002004683 | -- | |
| Unigene0033945 | 7.08 | 1.12E-05 | PREDICTED: 17.5 kDa class I heat shock protein-like [Cicer arietinum] | |
| Unigene0034025 | 10.34 | 0.002598569 | -- | |
| Unigene0034189 | 3.01 | 0.000124851 | PREDICTED: bax inhibitor 1-like [Arachis duranensis] | |
| Unigene0034435 | 6.22 | 0.011081909 | hypothetical protein MTR_3g083030 [Medicago truncatula] | |
| Unigene0034474 | 11.15 | 0.004918201 | -- | |
| Unigene0034546 | 4.64 | 0.000175208 | hsp20/alpha crystallin family protein [Medicago truncatula] | |
| Unigene0034873 | 2.87 | 0.000707212 | wound-responsive family protein [Medicago truncatula] | |
| Unigene0035006 | 2.71 | 0.006971265 | (2R)-phospho-3-sulfolactate synthase ComA [Medicago truncatula] | |
| Unigene0035057 | 3.96 | 0.000235239 | wound-responsive family protein [Medicago truncatula] | |
| Unigene0035081 | 5.33 | 0.003126452 | -- | |
| Unigene0035135 | 10.82 | 0.001479898 | PREDICTED: heat shock 70 kDa protein 15-like isoform X2 [Oryza brachyantha] | |
| Unigene0035177 | 6.26 | 0.042782194 | -- | |
| Unigene0035272 | 12.39 | 0.024728867 | 60S ribosomal protein L36 [Wolffia australiana] | |
| Unigene0035303 | 1.65 | 0.037078211 | protein phosphatase 2C-like protein [Medicago truncatula] | |
| Unigene0035396 | 13.51 | 0.008278186 | 40S ribosomal protein S14-3 [Morus notabilis] | |
| Unigene0036390 | 4.15 | 0.001033781 | hsp20/alpha crystallin family protein [Medicago truncatula] | |
| Unigene0036396 | 5.26 | 0.043140119 | ankyrin repeat plant-like protein [Medicago truncatula] | |
| Unigene0036508 | 11.98 | 0.0050088 | similar to S. cerevisiae PTR2 gene, GenBank Accession Number L11994 [Arabidopsis thaliana] | |
| Unigene0036679 | 6.00 | 0.011217616 | -- | |
| Unigene0036828 | 12.39 | 9.93E-07 | Ulp1 protease family, carboxy-terminal domain protein [Medicago truncatula] | |
| Unigene0036975 | 11.55 | 1.65E-05 | -- | |
| Unigene0037009 | 2.73 | 6.61E-05 | pre-mRNA-splicing factor SF2-like protein [Medicago truncatula] | |
| Unigene0037024 | 11.99 | 0.030279452 | hypothetical protein KFL_006750040 [Klebsormidium flaccidum] | |
| Unigene0037026 | 11.22 | 0.026040172 | -- | |
| Unigene0037472 | 3.58 | 0.003181292 | -- | |
| Unigene0037551 | 2.38 | 0.002465164 | rhomboid-like protein [Medicago truncatula] | |
| Unigene0037585 | 3.91 | 0.01946615 | hypothetical protein MTR_8g445380 [Medicago truncatula] | |
| Unigene0037788 | 2.77 | 0.00025082 | calcyclin-binding protein [Medicago truncatula] | |
| Unigene0037948 | 2.89 | 0.011174514 | wound-responsive family protein [Medicago truncatula] | |
| Unigene0038120 | 2.47 | 0.013305334 | PREDICTED: beta-glucosidase BoGH3B-like isoform X2 [Arachis duranensis] | |
| Unigene0038157 | 5.30 | 0.029552065 | general regulatory factor 2 [Medicago truncatula] | |
| Unigene0038284 | 7.03 | 0.014353925 | wall-associated kinase family protein [Medicago truncatula] | |
| Unigene0038485 | 4.91 | 0.038519159 | -- | |
| Unigene0038801 | 1.61 | 0.04954846 | COBRA-like protein 7 precursor [Medicago truncatula] | |
| Unigene0038822 | 1.50 | 0.045528272 | Serine/Threonine-kinase OXI1-like protein [Medicago truncatula] | |
| Unigene0038828 | 3.88 | 0.003363793 | class I heat shock protein, partial [Solanum nigrum] | |
| Unigene0039338 | 3.28 | 0.000967372 | DnaJ heat shock family protein [Medicago truncatula] | |
| Unigene0039591 | 6.96 | 0.039799433 | disease resistance protein (TIR-NBS-LRR class) [Medicago truncatula] | |
| Unigene0039675 | 10.21 | 0.006328811 | PREDICTED: chitotriosidase-1-like [Daucus carota subsp. sativus] [Daucus carota] | |
| Unigene0039855 | 4.22 | 5.79E-05 | localized small heat shock protein [Medicago truncatula] | |
| Unigene0040127 | 3.43 | 0.030095739 | RNA-directed DNA polymerase (Reverse transcriptase), Ribonuclease H-like protein [Theobroma cacao] | |
| Unigene0040422 | 1.93 | 0.030279452 | DnaJ heat shock amino-terminal domain protein [Medicago truncatula] | |
| Unigene0040431 | 11.55 | 0.025123832 | -- | |
| Unigene0040481 | 3.78 | 0.005596354 | ATP-dependent chaperone ClpB [Medicago truncatula] | |
| Unigene0040722 | 6.87 | 0.000900574 | -- | |
| Unigene0041383 | 13.32 | 0.015033921 | ribosomal protein S9, component of cytosolic 80S ribosome and 40S small subunit [Chlamydomonas reinhardtii] | |
| Unigene0041464 | 13.87 | 0.037494188 | disease resistance protein (TIR-NBS-LRR class) [Medicago truncatula] | |
| Unigene0041938 | 1.83 | 0.032259594 | heat shock protein 81-2 [Medicago truncatula] | |
| Unigene0042040 | 6.34 | 0.035551406 | -- | |
| Unigene0042075 | 3.05 | 0.02812805 | Lipid transfer protein [Medicago truncatula] | |
| Unigene0042112 | 10.02 | 0.02967127 | RNA-directed DNA polymerase , related [Medicago truncatula] | |
| Unigene0042117 | 9.76 | 0.031138073 | RNA-directed DNA polymerase (Reverse transcriptase) [Medicago truncatula] | |
| Unigene0042610 | 1.73 | 0.0123168 | splicing factor U2af small subunit A-like protein [Medicago truncatula] | |
| Unigene0042613 | 1.79 | 0.049339038 | glutathione S-transferase, amino-terminal domain protein [Medicago truncatula] | |
| Unigene0042682 | 10.63 | 0.015194598 | -- | |
| Unigene0042777 | 3.18 | 0.007691708 | Myb/SANT-like DNA-binding domain protein [Medicago truncatula] | |
| Unigene0042995 | 2.12 | 0.00882385 | PREDICTED: chaperone protein ClpB4, mitochondrial [Cicer arietinum] | |
| Unigene0043055 | 11.85 | 0.002341941 | RNA recognition motif [Medicago truncatula] | |
| Unigene0043325 | 3.50 | 0.026040172 | seed specific protein Bn15D17A [Medicago truncatula] | |
| Unigene0043681 | 1.95 | 0.038408719 | DnaJ heat shock amino-terminal domain protein [Medicago truncatula] | |
| Unigene0043700 | 3.56 | 0.002993739 | 7-dehydrocholesterol reductase [Medicago truncatula] | |
| Unigene0044103 | 3.22 | 0.038656231 | hypothetical protein MTR_5g006350 [Medicago truncatula] | |
| Unigene0044266 | 11.79 | 0.034810324 | disease resistance protein (TIR-NBS-LRR class) [Medicago truncatula] | |
| Unigene0044275 | 4.04 | 0.022228345 | PREDICTED: 70 kDa peptidyl-prolyl isomerase-like [Cicer arietinum] | |
| Unigene0044308 | 6.50 | 0.027773665 | disease resistance protein (TIR-NBS-LRR class) [Medicago truncatula] | |
| Unigene0044322 | 1.74 | 0.040692042 | DUF1685 family protein [Medicago truncatula] | |
| Unigene0044345 | 3.00 | 0.00788769 | 17.6 kDa class I heat shock protein [Medicago truncatula] | |
| Unigene0044624 | 2.39 | 0.01596238 | zinc-binding alcohol dehydrogenase family protein [Medicago truncatula] | |
| Unigene0045156 | 2.89 | 0.03458962 | Retrovirus-related Pol polyprotein from transposon opus [Cajanus cajan] | |
| Unigene0045388 | 8.00 | 0.001880743 | wall-associated receptor kinase-like protein [Medicago truncatula] | |
| Unigene0045409 | 4.97 | 0.016877833 | -- | |
| Unigene0045471 | 2.63 | 0.005378002 | -- | |
| Unigene0045533 | 2.39 | 0.001740805 | receptor family ligand-binding region protein [Medicago truncatula] | |
| Unigene0045891 | 2.73 | 0.021333876 | 17.6 kDa class I heat shock protein [Medicago truncatula] | |
| Unigene0045893 | 3.65 | 0.009827179 | heat shock protein [Medicago sativa] | |
| Unigene0045895 | 2.81 | 0.031213513 | heat shock protein [Medicago sativa] | |
| Unigene0045898 | 4.82 | 0.002568075 | PREDICTED: class I heat shock protein, partial [Nicotiana sylvestris] | |
| Unigene0045947 | 4.83 | 0.028017876 | -- | |
| Unigene0047227 | 3.39 | 8.17E-05 | heat shock transcription factor A3 [Medicago truncatula] | |
| Unigene0047313 | 1.95 | 0.009494267 | Ubiquitin fusion degradation protein 1 isogeny [Cajanus cajan] | |
| Unigene0047374 | 1.74 | 0.021118475 | transmembrane protein [Arabidopsis thaliana] | |
| Unigene0047640 | 4.40 | 0.0061514 | heat shock 70 kDa protein [Medicago truncatula] | |
| Unigene0047846 | 1.84 | 0.016460947 | Ulp1 protease family, carboxy-terminal domain protein [Medicago truncatula] | |
| Unigene0047898 | 6.50 | 0.026877924 | tubby-F-box-like protein [Medicago truncatula] | |
| Unigene0047943 | 2.40 | 0.022251733 | heat shock transcription factor A3 [Medicago truncatula] | |
| Unigene0048270 | 12.86 | 0.042398666 | histone h4, partial [Nannochloropsis gaditana CCMP526] | |
| Unigene0048279 | 2.15 | 0.000996378 | pyridine nucleotide-disulfide oxidoreductase family protein [Medicago truncatula] | |
| Unigene0048357 | 5.02 | 0.00173883 | wall-associated kinase family protein [Medicago truncatula] | |
| Unigene0048359 | 5.01 | 0.046536457 | wall-associated kinase family protein [Medicago truncatula] | |
| Unigene0048583 | 11.98 | 0.041837285 | elongation translation factor 1 alpha [Cyanophora paradoxa] | |
| Unigene0048584 | 12.31 | 0.020277105 | translation elongation factor 1-alpha, partial [Tengia sp. MEN-2015] | |
| Unigene0048585 | 12.50 | 0.005509849 | elongation factor 1-alpha [Chara braunii] | |
| Unigene0048682 | 3.37 | 0.000640097 | F-box plant-like protein [Medicago truncatula] | |
| Unigene0048970 | 12.79 | 0.011152311 | disease resistance protein (TIR-NBS-LRR class) [Medicago truncatula] | |
| Unigene0049012 | 3.19 | 7.69E-06 | heat shock 70 kDa protein [Medicago truncatula] | |
| Unigene0049082 | 11.59 | 0.041414718 | PREDICTED: protein SGT1 homolog A-like [Jatropha curcas] | |
| Unigene0049422 | 4.83 | 0.028004499 | -- | |
| Unigene0050034 | 12.72 | 0.019489401 | 40S ribosomal protein S17 [Auxenochlorella protothecoides] | |
| Unigene0050121 | 4.59 | 0.006371046 | -- | |
| Unigene0050391 | 11.99 | 5.82E-05 | -- | |
| Unigene0050824 | 7.48 | 0.008183096 | -- | |
| Unigene0051069 | 4.20 | 0.046968443 | -- | |
| Unigene0051126 | 12.57 | 0.017443691 | unknown [Populus trichocarpa x Populus deltoides] | |
| Unigene0051196 | 2.56 | 0.005960532 | PREDICTED: TMV resistance protein N-like [Cicer arietinum] | |
| Unigene0051515 | 3.54 | 0.009494267 | -- | |
| Unigene0051641 | 3.90 | 0.003939693 | Copia protein [Cajanus cajan] | |
| Unigene0051848 | 3.34 | 3.66E-05 | peptidyl-prolyl cis-trans isomerase FKBP62-like protein [Medicago truncatula] | |
| Unigene0052083 | 2.82 | 0.003595106 | PREDICTED: protein RFT1 homolog [Cicer arietinum] | |
| Unigene0052087 | 10.59 | 0.019958062 | nuclear division RFT1-like protein [Medicago truncatula] | |
| Unigene0052144 | 4.69 | 5.75E-06 | sterol regulatory element-binding protein site 2 protease [Medicago truncatula] | |
| Unigene0052292 | 2.86 | 0.000115339 | Erythroid differentiation-related factor 1 [Theobroma cacao] | |
| Unigene0052957 | 11.07 | 0.039964574 | -- | |
| Unigene0052963 | 5.66 | 0.021285708 | tyrosine kinase family protein [Medicago truncatula] | |
| Unigene0053063 | 10.80 | 0.004676128 | -- | |
| Unigene0053681 | 1.83 | 0.035526624 | PREDICTED: activator of 90 kDa heat shock protein ATPase homolog 1 [Cicer arietinum] | |
| Unigene0053686 | 2.59 | 0.000592253 | OPI10-like protein [Medicago truncatula] | |
| Unigene0053719 | 12.49 | 0.024735161 | aspartic protease family protein, putative [Medicago truncatula] | |
| Unigene0053728 | 7.21 | 0.017967993 | -- | |
| Unigene0054117 | 1.74 | 0.011130628 | C4-dicarboxylate transporter/malic acid transporter [Medicago truncatula] | |
| Unigene0054431 | 3.91 | 0.026040172 | Gag-pol polyprotein-like protein [Theobroma cacao] | |
| Unigene0054766 | 4.59 | 0.001657229 | -- | |
| Unigene0054921 | 6.46 | 0.015828635 | MFS transporter [Medicago truncatula] | |
| Unigene0054995 | 6.32 | 0.023390708 | senescence-associated protein [Medicago truncatula] | |
| Unigene0055143 | 10.95 | 0.002890743 | replication factor-A carboxy-terminal domain protein [Medicago truncatula] | |
| Unigene0055269 | 3.43 | 0.015194598 | BAG domain protein [Medicago truncatula] | |
| Unigene0055271 | 5.57 | 0.049496927 | BAG domain protein [Medicago truncatula] | |
| Unigene0055397 | 12.70 | 0.032025317 | PREDICTED: protein NRDE2 homolog isoform X3 [Cicer arietinum] | |
| Unigene0055478 | 3.29 | 0.001127994 | casein lytic proteinase B3 [Medicago truncatula] | |
| Unigene0055599 | 8.45 | 0.015278708 | -- | |
| Unigene0055700 | 5.17 | 0.011188729 | -- | |
| Unigene0055954 | 6.40 | 0.039307803 | LRR and NB-ARC domain disease resistance protein [Medicago truncatula] | |
| Unigene0055966 | 12.68 | 0.044761345 | hypothetical protein MTR_4g036475 [Medicago truncatula] | |
| Unigene0056188 | 13.00 | 0.005596354 | hAT dimerization domain-containing protein [Arabidopsis thaliana] | |
| Unigene0056283 | 8.61 | 0.014494493 | PREDICTED: glyceraldehyde-3-phosphate dehydrogenase GAPCP1, chloroplastic-like [Brassica oleracea var. oleracea] [Brassica oleracea] | |
| Unigene0056298 | 11.69 | 0.014494493 | PREDICTED: 3-isopropylmalate dehydrogenase-like [Ziziphus jujuba] | |
| Unigene0056323 | 10.77 | 0.004935451 | PREDICTED: chaperone protein ClpB1 [Citrus sinensis] | |
| Unigene0056364 | 12.33 | 0.011089935 | -- | |
| Unigene0056390 | 10.57 | 0.03016159 | duf89 fructose bisphosphatase [Nannochloropsis gaditana] | |
| Unigene0056427 | 9.79 | 0.006916976 | PREDICTED: protein NMT1 homolog [Pyrus x bretschneideri] | |
| Unigene0056445 | 11.90 | 0.008183096 | -- | |
| Unigene0056447 | 12.15 | 0.004531815 | PREDICTED: sugar transport protein 6-like [Nelumbo nucifera] | |
| Unigene0056487 | 11.33 | 0.004487042 | Transposon Ty3-I Gag-Pol polyprotein [Cajanus cajan] | |
| Unigene0056515 | 10.49 | 0.005547824 | -- | |
| Unigene0056552 | 13.01 | 0.012347801 | -- | |
| Unigene0056633 | 9.00 | 0.021118475 | 6-phosphogluconate dehydrogenase [Galdieria sulphuraria] | |
| Unigene0056673 | 11.91 | 0.025171254 | -- | |
| Unigene0056712 | 9.03 | 0.019264629 | PREDICTED: 60S ribosomal protein L17-1-like [Gossypium arboreum] | |
| Unigene0056772 | 11.27 | 0.025365709 | Metalloenzyme, LuxS/M16 peptidase-like [Ostreococcus tauri] | |
| Unigene0056777 | 11.75 | 0.009541475 | eukaryotic initiation factor 4a [Nannochloropsis gaditana] | |
| Unigene0056815 | 7.97 | 0.006729683 | -- | |
| Unigene0056852 | 12.75 | 0.032933629 | ribosomal protein L14b/L23e [Coccomyxa subellipsoidea C-169] | |
| Unigene0056890 | 10.99 | 0.041195701 | myo-inositol-1-phosphate synthase [Chlamydomonas reinhardtii] | |
| Unigene0057005 | 12.34 | 0.021932245 | -- | |
| Unigene0057034 | 10.54 | 0.014698166 | -- | |
| Unigene0057177 | 13.71 | 0.013194965 | 60S ribosomal protein L12 [Zea mays] | |
| Unigene0057222 | 12.86 | 0.028865076 | 40S ribosomal protein S21 [Oxyrrhis marina] | |
| Unigene0057498 | 13.39 | 0.012043626 | -- | |
| Unigene0057536 | 14.06 | 0.007448122 | PREDICTED: 60S ribosomal protein L11-like [Phoenix dactylifera] | |
| Unigene0057554 | 13.08 | 0.006661832 | receptor for activated protein kinase C [Chondrus crispus] | |
| Unigene0057571 | 11.15 | 0.034610551 | PREDICTED: BI1-like protein [Populus euphratica] | |
| Unigene0057583 | 11.82 | 0.006474152 | -- | |
| Unigene0057590 | 11.26 | 0.037423884 | 20S core proteasome subunit beta 5 [Chondrus crispus] | |
| Unigene0057612 | 13.52 | 0.035551406 | histone H2B [Micromonas commoda] | |
| Unigene0057630 | 11.94 | 0.009577835 | delta12 fatty acid desaturase [Chondrus crispus] | |
| Unigene0057644 | 10.05 | 0.031928066 | Retrotransposable element Tf2 [Cajanus cajan] | |
| Unigene0057667 | 10.88 | 0.032530081 | PREDICTED: cell division control protein 3-like [Pyrus x bretschneideri] | |
| Unigene0057671 | 11.35 | 0.043282983 | -- | |
| Unigene0057710 | 13.41 | 0.006932325 | -- | |
| Unigene0057721 | 13.54 | 0.008395505 | PREDICTED: 60S ribosomal protein L8-1-like [Ziziphus jujuba] | |
| Unigene0057733 | 12.55 | 0.012172516 | malate dehydrogenase [Volvox carteri f. nagariensis] | |
| Unigene0057770 | 12.03 | 0.003678092 | cell division cycle protein 48 homolog [Glycine max] | |
| Unigene0057771 | 9.94 | 0.004487845 | PREDICTED: chitotriosidase-1-like [Brassica napus] | |
| Unigene0057784 | 10.95 | 0.047893631 | -- | |
| Unigene0057804 | 13.78 | 0.00426066 | -- | |
| Unigene0057814 | 11.89 | 0.028720945 | -- | |
| Unigene0057928 | 12.30 | 0.047893631 | -- | |
| Unigene0057957 | 10.84 | 0.042398666 | PREDICTED: L-ascorbate oxidase-like [Vigna radiata var. radiata] [Vigna radiata] | |
| Unigene0057972 | 11.23 | 0.039831288 | -- | |
| Unigene0057973 | 13.47 | 0.016667945 | ribosomal protein S27-like protein [Solanum tuberosum] | |
| Unigene0058012 | 14.82 | 0.001479898 | peptidylprolyl isomerase [Galdieria sulphuraria] | |
| Unigene0058081 | 11.78 | 0.03484521 | SKP1-like protein ASK10, partial [Arabidopsis thaliana] | |
| Unigene0058095 | 11.47 | 0.022228345 | Polynucleotidyl transferase, Ribonuclease H fold [Medicago truncatula] | |
| Unigene0058148 | 14.40 | 0.008103827 | component of cytosolic 80S ribosome and 40S small subunit [Volvox carteri f. nagariensis] | |
| Unigene0058255 | 12.81 | 0.00788769 | PREDICTED: nuclear transport factor 2 [Musa acuminata subsp. malaccensis] [Musa acuminata] | |
| Unigene0058345 | 11.12 | 0.014171769 | -- | |
| Unigene0058445 | 13.81 | 0.04952697 | -- | |
| Unigene0058456 | 13.31 | 0.006368653 | 60s ribosomal protein l20 [Chrysochromulina sp. CCMP291] | |
| Unigene0058520 | 12.57 | 0.045964689 | large subunit ribosomal protein L38e_1, cytoplasmic, partial [Guillardia theta CCMP2712] | |
| Unigene0058716 | 11.90 | 0.005492379 | MFS transporter, SP family, sugar:H+ symporter [Galdieria sulphuraria] | |
| Unigene0058721 | 11.16 | 0.013348202 | succinate dehydrogenase flavoprotein [Phaeodactylum tricornutum CCAP 1055/1] | |
| Unigene0058731 | 13.23 | 0.005549865 | PREDICTED: uncharacterized protein OsI_027940-like isoform X1 [Solanum pennellii] | |
| Unigene0058761 | 13.49 | 0.005596354 | -- | |
| Unigene0058769 | 10.99 | 0.013332943 | PREDICTED: uncharacterized zinc finger protein At4g06634-like [Oryza brachyantha] | |
| Unigene0058776 | 7.39 | 0.041785293 | PREDICTED: subtilisin-like protease [Sesamum indicum] | |
| Unigene0058794 | 13.59 | 0.002890743 | -- | |
| Unigene0058798 | 13.54 | 0.004487042 | PREDICTED: 40S ribosomal protein S3-1-like [Beta vulgaris subsp. vulgaris] [Beta vulgaris] | |
| Unigene0058802 | 10.48 | 0.018963097 | Ribonuclease H, partial [Medicago truncatula] | |
| Unigene0058808 | 13.05 | 0.019980397 | large subunit ribosomal protein L22e [Nannochloropsis gaditana CCMP526] | |
| Unigene0058814 | 13.30 | 0.01790718 | 60S ribosomal protein L28-1 [Monoraphidium neglectum] | |
| Unigene0058874 | 10.74 | 0.030283622 | predicted protein [Hordeum vulgare subsp. vulgare] [Hordeum vulgare] | |
| Unigene0058877 | 8.97 | 0.021879615 | ribosomal protein L13e [Helicosporidium sp. ATCC 50920] | |
| Unigene0058918 | 8.03 | 0.049496927 | translationally-controlled tumor protein homolog [Zea mays] | |
| Unigene0058996 | 11.19 | 0.010607994 | -- | |
| Unigene0059005 | 11.57 | 0.040791389 | -- | |
| Unigene0059036 | 11.78 | 0.012351216 | cycloartenol-C24-methyltransferase [Oryza sativa Japonica Group] | |
| Unigene0059077 | 11.02 | 0.044488151 | endo-1,4-beta-mannosidase [Klebsormidium flaccidum] | |
| Unigene0059085 | 12.89 | 0.024728867 | 60S ribosomal protein L37 [Chondrus crispus] | |
| Unigene0059102 | 14.27 | 0.002134277 | -- | |
| Unigene0059399 | 11.63 | 0.021605363 | PREDICTED: small glutamine-rich tetratricopeptide repeat-containing protein [Brassica rapa] | |
| Unigene0059440 | 13.02 | 0.008437055 | Glutathione S-transferase [Chondrus crispus] | |
| Unigene0059483 | 12.36 | 0.018752059 | -- | |
| Unigene0059959 | 11.62 | 0.038614264 | calmodulin [Zea mays] | |
| Unigene0059966 | 8.64 | 0.028324121 | enolase [Chlamydomonas reinhardtii] | |
| Unigene0059971 | 11.62 | 0.030463833 | PREDICTED: reactive Intermediate Deaminase A, chloroplastic-like [Malus domestica] | |
| Unigene0059981 | 13.47 | 0.005852769 | -- | |
| Unigene0059986 | 11.11 | 0.019264629 | PREDICTED: lysophospholipid acyltransferase 1 [Tarenaya hassleriana] | |
| Unigene0060005 | 10.82 | 0.030978859 | PREDICTED: copia protein isoform X2 [Beta vulgaris subsp. vulgaris] [Beta vulgaris] | |
| Unigene0060025 | 8.62 | 0.036933158 | PREDICTED: 60S ribosomal protein L15-1-like [Tarenaya hassleriana] | |
| Unigene0060042 | 13.39 | 0.007249236 | PREDICTED: 40S ribosomal protein S18 [Setaria italica] | |
| Unigene0060043 | 12.51 | 0.009926252 | L-ascorbate peroxidase [Volvox carteri f. nagariensis] | |
| Unigene0060046 | 13.52 | 0.003826191 | 40S ribosomal protein S4 [Zea mays] | |
| Unigene0060071 | 11.83 | 0.019599588 | -- | |
| Unigene0060077 | 10.20 | 0.005044898 | -- | |
| Unigene0060104 | 12.25 | 0.025171254 | -- | |
| Unigene0060106 | 9.53 | 0.003204823 | -- | |
| Unigene0060117 | 8.79 | 0.00882385 | RNA-binding protein [Emiliania huxleyi CCMP1516] | |
| Unigene0060139 | 8.19 | 0.010692781 | PREDICTED: uncharacterized protein LOC101494553 [Cicer arietinum] | |
| Unigene0060167 | 11.34 | 0.035612084 | unknown [Populus trichocarpa x Populus deltoides] | |
| Unigene0060177 | 9.64 | 0.013412075 | F-type H+-transporting ATPase subunit beta [Galdieria sulphuraria] | |
| Unigene0060189 | 12.34 | 0.025365709 | 14 kDa subunit of cytochrome bd ubiquinol oxidase [Coccomyxa subellipsoidea C-169] | |
| Unigene0060192 | 11.94 | 0.011160209 | PREDICTED: pyruvate dehydrogenase E1 component subunit alpha, mitochondrial [Vigna angularis] | |
| Unigene0060196 | 11.26 | 0.025914156 | PREDICTED: probable mediator of RNA polymerase II transcription subunit 36b isoform X1 [Brassica napus] | |
| Unigene0060199 | 11.36 | 0.013065209 | Acetyl-CoA acetyltransferase [Ectocarpus siliculosus] | |
| Unigene0060202 | 11.19 | 0.044435971 | Retrotransposable element Tf2 [Cajanus cajan] | |
| Unigene0060209 | 11.10 | 0.035073564 | GDP-mannose pyrophosphorylase [Klebsormidium flaccidum] | |
| Unigene0060219 | 12.80 | 0.010266886 | transaldolase [Thalassiosira pseudonana CCMP1335] | |
| Unigene0060221 | 11.79 | 0.000619904 | -- | |
| Unigene0060227 | 12.29 | 0.014691078 | H+-transporting two-sector ATPase, alpha/beta subunit, central region, related [Medicago truncatula] | |
| Unigene0060247 | 13.51 | 0.012708308 | PREDICTED: 60S ribosomal protein L10-like isoform X2 [Pyrus x bretschneideri] | |
| Unigene0060249 | 10.93 | 0.026401935 | PREDICTED: pre-mRNA-processing factor 19 homolog 2 [Camelina sativa] | |
| Unigene0060321 | 14.50 | 0.003889718 | histone H2B [Micromonas commoda] | |
| Unigene0060334 | 13.02 | 0.004231481 | peptidyl-prolyl cis-trans isomerase [Klebsormidium flaccidum] | |
| Unigene0060343 | 11.09 | 0.031143175 | proliferation-associated protein 1 [Coccomyxa subellipsoidea C-169] | |
| Unigene0060379 | 11.91 | 0.046068717 | hypothetical protein AURANDRAFT_62014 [Aureococcus anophagefferens] | |
| Unigene0060417 | 7.96 | 0.01596238 | -- | |
| Unigene0060470 | 12.20 | 0.020121576 | PREDICTED: 60S ribosomal protein L35a-1 [Pyrus x bretschneideri] | |
| Unigene0060611 | 11.31 | 0.045215004 | 20S proteasome subunit alpha type 3 [Guillardia theta CCMP2712] | |
| Unigene0060760 | 9.22 | 0.013230476 | polyubiquitin, partial [Cicer arietinum] | |
| Unigene0061220 | 11.77 | 0.037887511 | Ubiquitin-conjugating enzyme [Plantago major] | |
| Unigene0061226 | 11.92 | 0.004676128 | -- | |
| Unigene0061230 | 10.91 | 0.037223895 | -- | |
| Unigene0061235 | 14.03 | 0.004263413 | 40S ribosomal protein S26 [Zea mays] | |
| Unigene0061255 | 11.50 | 0.035155605 | trehalose-phosphate synthase 6 [Camellia sinensis] | |
| Unigene0061268 | 13.14 | 0.006467823 | PREDICTED: 60S ribosomal protein L1-A [Pyrus x bretschneideri] | |
| Unigene0061279 | 9.82 | 0.010197078 | PREDICTED: 60S acidic ribosomal protein P0-like [Glycine max] | |
| Unigene0061285 | 11.84 | 0.021751875 | PREDICTED: nucleolin 2-like [Elaeis guineensis] | |
| Unigene0061289 | 10.48 | 0.000640097 | pectin methylesterase [Vitis riparia] | |
| Unigene0061328 | 13.66 | 0.007216297 | 60S ribosomal protein L5-2 [Zea mays] | |
| Unigene0061330 | 13.16 | 0.007631584 | ribosomal protein L32, component of cytosolic 80S ribosome and 60S large subunit [Chlamydomonas reinhardtii] | |
| Unigene0061338 | 12.99 | 0.003889718 | Glutamate ammonia ligase [Ectocarpus siliculosus] | |
| Unigene0061355 | 10.85 | 0.033920948 | Heat shock protein 70, conserved site-containing protein, partial [Cynara cardunculus var. scolymus] [Cynara cardunculus] | |
| Unigene0061388 | 13.74 | 0.0061514 | PREDICTED: 40S ribosomal protein S15-like [Phoenix dactylifera] | |
| Unigene0061402 | 8.37 | 0.043282983 | 40S ribosomal protein S11e [Galdieria sulphuraria] | |
| Unigene0061410 | 10.48 | 0.046824822 | -- | |
| Unigene0061416 | 13.83 | 0.001880743 | -- | |
| Unigene0061466 | 11.35 | 0.018183208 | PREDICTED: guanosine nucleotide diphosphate dissociation inhibitor At5g09550 [Jatropha curcas] | |
| Unigene0061570 | 14.57 | 0.002150944 | 2,3-dihydro-2,3-dihydroxybenzoate dehydrogenase [Cyanidioschyzon merolae strain 10D] [Cyanidioschyzon merolae] | |
| Unigene0061623 | 8.14 | 0.036250365 | acetohydroxyacid isomeroreductase [Glycine max] | |
| Unigene0061625 | 10.80 | 0.03136078 | -- | |
| Unigene0061641 | 11.23 | 0.044141168 | unknown [Populus trichocarpa x Populus deltoides] | |
| Unigene0061647 | 11.13 | 0.035421077 | Succinyl-CoA Synthetase subunit ? (SCS?) [Blastocystis hominis] | |
| Unigene0061668 | 14.24 | 0.006521489 | PREDICTED: histone H2A.6-like [Daucus carota subsp. sativus] [Daucus carota] | |
| Unigene0061675 | 10.73 | 0.032884552 | Basic leucine zipper and W2 domain-containing protein 2 [Ananas comosus] | |
| Unigene0061787 | 12.88 | 0.026992032 | -- | |
| Unigene0061877 | 13.60 | 0.008796077 | PREDICTED: ubiquitin-40S ribosomal protein S27a-2-like [Camelina sativa] | |
| Unigene0062406 | 12.17 | 0.008014944 | molecular chaperone DnaK [Galdieria sulphuraria] | |
| Unigene0062413 | 11.75 | 0.01565511 | phosphoglycerate kinase [Chondrus crispus] | |
| Unigene0062424 | 13.24 | 0.019133271 | 60S ribosomal protein L30 [Klebsormidium flaccidum] | |
| Unigene0062457 | 11.20 | 0.019822935 | -- | |
| Unigene0062461 | 11.82 | 0.002896205 | 1a protein [Cucumber mosaic virus] | |
| Unigene0062464 | 8.30 | 0.039831288 | -- | |
| Unigene0062512 | 11.90 | 0.035187016 | TPA: histone H3 [Zea mays] | |
| Unigene0062513 | 9.08 | 0.004676128 | -- | |
| Unigene0062525 | 13.05 | 0.005237014 | PREDICTED: 60S ribosomal protein L23a-like [Cucumis sativus] | |
| Unigene0062527 | 9.89 | 0.004425198 | -- | |
| Unigene0062542 | 10.69 | 0.025914156 | -- | |
| Unigene0062545 | 11.67 | 0.018975927 | Transposon Ty3-I Gag-Pol polyprotein [Cajanus cajan] | |
| Unigene0062548 | 11.03 | 0.036382225 | -- | |
| Unigene0062556 | 13.22 | 0.033385471 | -- | |
| Unigene0062563 | 11.87 | 0.048288328 | PREDICTED: bax inhibitor 1-like [Musa acuminata subsp. malaccensis] [Musa acuminata] | |
| Unigene0062599 | 10.25 | 0.007469747 | Protein PRY1 [Monoraphidium neglectum] | |
| Unigene0062602 | 13.08 | 0.013324709 | -- | |
| Unigene0062626 | 11.44 | 0.026992032 | PREDICTED: L-idonate 5-dehydrogenase-like isoform X2 [Vitis vinifera] | |
| Unigene0062652 | 12.35 | 0.031271708 | -- | |
| Unigene0062672 | 11.27 | 0.043760095 | 20S core proteasome subunit alpha protein [Klebsormidium flaccidum] | |
| Unigene0062718 | 11.85 | 0.013305334 | cytosolic class II aldolase [Phaeodactylum tricornutum CCAP 1055/1] | |
| Unigene0062739 | 9.02 | 0.035688234 | -- | |
| Unigene0062754 | 14.10 | 0.025966004 | 60S acidic ribosomal protein P2B [Zea mays] | |
| Unigene0062765 | 10.74 | 0.001023062 | -- | |
| Unigene0062790 | 12.12 | 0.012536726 | PREDICTED: sorting nexin 1 isoform X2 [Citrus sinensis] | |
| Unigene0062812 | 11.13 | 0.026746844 | -- | |
| Unigene0062841 | 12.33 | 0.013829818 | PDIL2-2 - Zea mays protein disulfide isomerase [Zea mays] | |
| Unigene0062849 | 8.74 | 0.023440342 | molecular chaperone DnaJ [Galdieria sulphuraria] | |
| Unigene0062899 | 11.52 | 0.033659327 | arad-like aldolase/epimerase, partial [Coccomyxa subellipsoidea C-169] | |
| Unigene0062932 | 11.60 | 0.04400263 | transcription factor BTF3 [Zea mays] | |
| Unigene0062951 | 12.54 | 0.011409804 | -- | |
| Unigene0063052 | 10.85 | 0.040655182 | Cyclin-P3-1 [Zostera marina] | |
| Unigene0063080 | 14.17 | 0.004955878 | -- | |
| Unigene0063131 | 9.42 | 0.007197311 | -- | |
| Unigene0063386 | 14.16 | 0.019822935 | -- | |
| Unigene0063389 | 11.82 | 0.011174514 | aquaglyceroporin related protein, MIP family [Galdieria sulphuraria] | |
| Unigene0063535 | 10.81 | 0.000619904 | -- | |
| Unigene0063997 | 11.74 | 0.029964637 | Transposon Ty3-G Gag-Pol polyprotein [Cajanus cajan] | |
| Unigene0064020 | 8.21 | 0.039690891 | PREDICTED: DEAD-box ATP-dependent RNA helicase 56 isoform X1 [Fragaria vesca subsp. vesca] [Fragaria vesca] | |
| Unigene0064028 | 13.55 | 0.005549865 | PREDICTED: 40S ribosomal protein S16 [Solanum lycopersicum] | |
| Unigene0064029 | 11.20 | 0.031268601 | hypothetical protein COCSUDRAFT_47264 [Coccomyxa subellipsoidea C-169] | |
| Unigene0064076 | 11.42 | 0.026746844 | PREDICTED: protein BROTHER of FT and TFL 1-like [Camelina sativa] | |
| Unigene0064187 | 11.17 | 0.026746844 | -- | |
| Unigene0064234 | 13.87 | 0.010197078 | histone H2A [Physcomitrella patens] | |
| Unigene0064274 | 11.20 | 0.021118475 | PREDICTED: serine/arginine-rich splicing factor SR45a-like [Brassica rapa] | |
| Unigene0064564 | 12.59 | 0.014132216 | -- | |
| Unigene0064693 | 13.61 | 0.007028841 | PREDICTED: 14-3-3-like protein D [Elaeis guineensis] | |
| Unigene0064746 | 12.02 | 0.048402456 | -- | |
| Unigene0064795 | 11.57 | 0.014129366 | similar to bleomycin hydrolase [Ectocarpus siliculosus] | |
| Unigene0064894 | 6.02 | 0.032530081 | Retrovirus-related Pol polyprotein from transposon TNT 1-94 [Cajanus cajan] | |
| Unigene0065106 | 10.33 | 0.032634054 | heat shock protein ClpB [Cyanidioschyzon merolae strain 10D] [Cyanidioschyzon merolae] | |
| Unigene0065134 | 11.05 | 0.024735161 | PREDICTED: 3-ketoacyl-CoA thiolase 2, peroxisomal-like [Arachis duranensis] | |
| Unigene0065179 | 13.43 | 0.005237014 | -- | |
| Unigene0065221 | 7.08 | 0.043528616 | hypothetical protein TSUD_284280 [Trifolium subterraneum] | |
| Unigene0065308 | 10.65 | 0.030556219 | glycerol-3-phosphate dehydrogenase [Galdieria sulphuraria] | |
| Unigene0065366 | 13.15 | 0.008890411 | 60S ribosomal protein L7 family protein [Klebsormidium flaccidum] | |
| Unigene0065475 | 11.95 | 0.033659327 | -- | |
| Unigene0065680 | 11.48 | 0.037911145 | -- | |
| Unigene0065681 | 13.02 | 0.000714691 | PREDICTED: 14-3-3-like protein GF14 psi [Brassica rapa] | |
| Unigene0065689 | 10.79 | 0.030270637 | PREDICTED: cytochrome P450 94B3-like [Solanum pennellii] | |
| Unigene0065731 | 10.97 | 0.04121937 | glycoside hydrolase family 76 [Chrysochromulina sp. CCMP291] | |
| Unigene0066006 | 5.79 | 0.011081909 | BnaAnng16530D [Brassica napus] | |
| Unigene0066076 | 11.38 | 0.032933629 | -- | |
| Unigene0066195 | 10.91 | 0.017642327 | RNA-directed DNA polymerase (Reverse transcriptase) [Medicago truncatula] | |
| Unigene0066276 | 10.26 | 0.006328811 | PREDICTED: ADP,ATP carrier protein 1, mitochondrial [Oryza sativa Japonica Group] | |
| Unigene0066280 | 10.72 | 0.020225406 | ribonuclease II family protein [Klebsormidium flaccidum] | |
| Unigene0066299 | 9.89 | 0.006474152 | -- | |
| Unigene0066328 | 8.57 | 0.028324121 | 60S acidic ribosomal family protein [Arabidopsis thaliana] | |
| Unigene0066362 | 10.87 | 0.018369759 | -- | |
| Unigene0066423 | 7.56 | 0.043445764 | 40S ribosomal protein-like protein [Thalassiosira pseudonana CCMP1335] | |
| Unigene0066643 | 13.63 | 0.007249236 | PREDICTED: 40S ribosomal protein S24-1-like [Fragaria vesca subsp. vesca] [Fragaria vesca] | |
| Unigene0000033 | -1.74 | 0.008497118 | beta-like galactosidase [Medicago truncatula] |  |
| Unigene0000164 | -5.06 | 0.037423884 | RNA-directed DNA polymerase (Reverse transcriptase); Ribonuclease H; Endonuclease/exonuclease/phosphatase [Medicago truncatula] |  |
| Unigene0000203 | -2.55 | 0.045323595 | F-box protein MAX2 [Medicago truncatula] |  |
| Unigene0000663 | -2.34 | 0.045514801 | PREDICTED: stress response protein nst1 [Vigna angularis] |  |
| Unigene0000830 | -2.07 | 0.030939539 | NBS1-like protein [Medicago truncatula] |  |
| Unigene0001255 | -4.60 | 0.034028894 | transmembrane protein, putative [Medicago truncatula] |  |
| Unigene0001272 | -3.65 | 0.006328811 | ethylene-responsive transcription factor ERF017-like protein [Medicago truncatula] |  |
| Unigene0001869 | -3.19 | 0.03668925 | cytochrome P450 family protein [Medicago truncatula] |  |
| Unigene0001912 | -1.78 | 0.032472202 | RNA-binding domain CCCH-type zinc finger protein [Medicago truncatula] |  |
| Unigene0001987 | -4.45 | 0.010993135 | Retrovirus-related Pol polyprotein from transposon 17.6, partial [Cajanus cajan] |  |
| Unigene0002039 | -3.71 | 0.037753713 | helix loop helix DNA-binding domain protein [Medicago truncatula] |  |
| Unigene0002071 | -4.55 | 0.001935815 | specific tissue protein [Medicago truncatula] |  |
| Unigene0002143 | -11.06 | 0.026746844 | albumin I [Medicago truncatula] |  |
| Unigene0002499 | -3.37 | 0.004599517 | F-box/kelch-repeat plant-like protein [Medicago truncatula] |  |
| Unigene0002788 | -3.63 | 0.031259607 | -- |  |
| Unigene0002855 | -2.04 | 0.046536457 | LRR receptor-like kinase [Medicago truncatula] |  |
| Unigene0002862 | -3.92 | 0.026382019 | receptor-like kinase feronia-like protein [Medicago truncatula] |  |
| Unigene0002876 | -2.16 | 0.033425478 | feronia receptor-like kinase [Medicago truncatula] |  |
| Unigene0002877 | -2.08 | 0.030270637 | feronia receptor-like kinase [Medicago truncatula] |  |
| Unigene0002878 | -3.27 | 0.006521489 | feronia receptor-like kinase [Medicago truncatula] |  |
| Unigene0002884 | -5.16 | 0.000117856 | receptor-like kinase feronia-like protein [Medicago truncatula] |  |
| Unigene0002893 | -4.77 | 0.021118475 | feronia receptor-like kinase [Medicago truncatula] |  |
| Unigene0002950 | -2.19 | 0.002996464 | LRR receptor-like kinase [Medicago truncatula] |  |
| Unigene0002968 | -4.54 | 0.044965767 | -- |  |
| Unigene0002983 | -11.71 | 0.003875774 | -- |  |
| Unigene0003130 | -3.26 | 2.92E-06 | transmembrane protein, putative [Medicago truncatula] |  |
| Unigene0003474 | -3.02 | 0.042661507 | LRR receptor-like kinase family protein [Medicago truncatula] |  |
| Unigene0003484 | -4.71 | 0.002473103 | LRR receptor-like kinase family protein [Medicago truncatula] |  |
| Unigene0003488 | -5.41 | 0.001932376 | LRR receptor-like kinase family protein [Medicago truncatula] |  |
| Unigene0003489 | -2.75 | 0.039799433 | LRR receptor-like kinase family protein [Medicago truncatula] |  |
| Unigene0003498 | -2.88 | 0.014353925 | LRR receptor-like kinase family protein [Medicago truncatula] |  |
| Unigene0003505 | -2.39 | 0.018687328 | LRR receptor-like kinase family protein [Medicago truncatula] |  |
| Unigene0003557 | -2.43 | 0.046434665 | disease resistance protein (TIR-NBS-LRR class) [Medicago truncatula] |  |
| Unigene0003680 | -3.71 | 0.004734971 | PREDICTED: TMV resistance protein N-like isoform X3 [Cicer arietinum] |  |
| Unigene0003824 | -4.51 | 0.009550139 | LRR receptor-like kinase plant [Medicago truncatula] |  |
| Unigene0004240 | -11.74 | 0.000203333 | -- |  |
| Unigene0004247 | -2.60 | 0.011409804 | disease resistance protein (TIR-NBS-LRR class) [Medicago truncatula] |  |
| Unigene0004252 | -2.12 | 0.025123832 | DNA replication factor CDT1-like protein [Medicago truncatula] |  |
| Unigene0004922 | -2.18 | 0.016843419 | pectinesterase/pectinesterase inhibitor [Medicago truncatula] |  |
| Unigene0004982 | -3.06 | 0.047471598 | ovate transcriptional repressor [Medicago truncatula] |  |
| Unigene0005052 | -10.84 | 0.011922083 | disease resistance-responsive, dirigent domain protein [Medicago truncatula] |  |
| Unigene0005129 | -11.96 | 0.019264629 | NB-ARC domain disease resistance protein [Medicago truncatula] |  |
| Unigene0005238 | -2.55 | 0.037223895 | exocyst subunit exo70 family protein [Medicago truncatula] |  |
| Unigene0005319 | -3.96 | 0.023814928 | -- |  |
| Unigene0005349 | -2.24 | 0.028855727 | receptor-like protein [Medicago truncatula] |  |
| Unigene0005351 | -2.42 | 0.031008908 | receptor-like protein [Medicago truncatula] |  |
| Unigene0005379 | -1.75 | 0.026297005 | cysteine-rich receptor-kinase-like protein [Medicago truncatula] |  |
| Unigene0005701 | -2.42 | 0.002993739 | heavy metal-associated domain protein [Medicago truncatula] |  |
| Unigene0005712 | -2.21 | 0.014494493 | wall-associated kinase family protein [Medicago truncatula] |  |
| Unigene0006314 | -10.54 | 0.041837285 | ATP-dependent RNA helicase A-like protein [Medicago truncatula] |  |
| Unigene0006463 | -2.57 | 0.006916976 | disease resistance protein (TIR-NBS-LRR class) [Medicago truncatula] |  |
| Unigene0006984 | -2.19 | 0.04471027 | wall-associated receptor kinase-like protein [Medicago truncatula] |  |
| Unigene0007117 | -1.62 | 0.030270637 | LRR receptor-like kinase family protein [Medicago truncatula] |  |
| Unigene0007278 | -3.72 | 0.034340573 | -- |  |
| Unigene0007363 | -10.60 | 0.004348684 | PREDICTED: zinc finger BED domain-containing protein DAYSLEEPER-like [Vitis vinifera] |  |
| Unigene0007410 | -12.89 | 1.37E-05 | -- |  |
| Unigene0007524 | -10.70 | 0.006328811 | -- |  |
| Unigene0007568 | -1.86 | 0.049496927 | xyloglucan endotransglucosylase/hydrolase family protein [Medicago truncatula] |  |
| Unigene0007585 | -6.69 | 0.004487042 | -- |  |
| Unigene0007747 | -10.10 | 0.038060396 | NBS-LRR resistance protein [Medicago truncatula] |  |
| Unigene0007865 | -12.73 | 0.000229002 | NBS-LRR type disease resistance protein [Medicago truncatula] |  |
| Unigene0007911 | -7.01 | 0.025914156 | -- |  |
| Unigene0007973 | -5.22 | 0.007278067 | NBS-LRR type disease resistance protein [Medicago truncatula] |  |
| Unigene0007975 | -12.20 | 2.96E-05 | -- |  |
| Unigene0008008 | -2.38 | 0.024216676 | tyrosine kinase family protein [Medicago truncatula] |  |
| Unigene0008201 | -4.46 | 1.77E-09 | sulfate transporter 3.1-like protein [Pisum sativum] |  |
| Unigene0008413 | -6.80 | 0.037494188 | NB-ARC domain protein [Medicago truncatula] |  |
| Unigene0008483 | -2.81 | 0.016844018 | DUF581 family protein [Medicago truncatula] |  |
| Unigene0008524 | -2.36 | 0.041195701 | S-locus lectin kinase family protein [Medicago truncatula] |  |
| Unigene0008542 | -2.03 | 0.032607048 | transcription factor HB29-like protein [Medicago truncatula] |  |
| Unigene0008598 | -12.20 | 0.011409804 | alpha/beta hydrolase family protein [Medicago truncatula] |  |
| Unigene0008685 | -2.27 | 0.009110915 | ethylene-responsive transcription factor ERF017-like protein [Medicago truncatula] |  |
| Unigene0008795 | -1.94 | 0.017184914 | PREDICTED: probable F-box protein At4g22030 [Cicer arietinum] |  |
| Unigene0008916 | -11.45 | 0.004676128 | Sad1/UNC-like carboxy-terminal protein [Medicago truncatula] |  |
| Unigene0008959 | -12.91 | 6.34E-06 | wall-associated receptor kinase galacturonan-binding protein [Medicago truncatula] |  |
| Unigene0008993 | -6.78 | 0.006774791 | transmembrane protein, putative [Medicago truncatula] |  |
| Unigene0009271 | -2.68 | 0.000743608 | zinc finger constans-like protein [Medicago truncatula] |  |
| Unigene0009315 | -5.17 | 0.003243407 | PREDICTED: N6-adenosine-methyltransferase MT-A70-like [Populus euphratica] |  |
| Unigene0009363 | -3.40 | 0.004676128 | UDP-glucosyltransferase family protein [Medicago truncatula] |  |
| Unigene0009757 | -3.27 | 0.005852769 | PREDICTED: methylthioribose kinase-like [Arachis ipaensis] |  |
| Unigene0009951 | -4.00 | 0.001747742 | PREDICTED: TMV resistance protein N-like isoform X1 [Cicer arietinum] |  |
| Unigene0010876 | -2.81 | 0.048288328 | PREDICTED: senescence-associated carboxylesterase 101-like isoform X2 [Cicer arietinum] |  |
| Unigene0010877 | -3.53 | 0.026242227 | PREDICTED: senescence-associated carboxylesterase 101-like isoform X2 [Cicer arietinum] |  |
| Unigene0011458 | -12.32 | 0.000104005 | -- |  |
| Unigene0011536 | -4.75 | 0.014907383 | -- |  |
| Unigene0011667 | -2.30 | 0.029552065 | disease resistance protein (TIR-NBS-LRR class) [Medicago truncatula] |  |
| Unigene0011685 | -11.14 | 0.032527889 | -- |  |
| Unigene0011802 | -6.33 | 0.033910093 | MYB transcription factor MYB51 [Medicago truncatula] |  |
| Unigene0011898 | -3.03 | 0.002438704 | cytochrome P450 family protein [Medicago truncatula] |  |
| Unigene0012197 | -2.46 | 0.01542643 | MFS transporter [Medicago truncatula] |  |
| Unigene0012382 | -2.96 | 0.016963663 | LRR receptor-like kinase family protein [Medicago truncatula] |  |
| Unigene0012433 | -11.11 | 0.010342681 | -- |  |
| Unigene0012800 | -11.22 | 0.004422821 | RING-H2 zinc finger protein [Medicago truncatula] |  |
| Unigene0013084 | -10.04 | 0.032968356 | DUF4283 domain protein [Medicago truncatula] |  |
| Unigene0013426 | -3.81 | 0.000247614 | zinc finger constans-like protein [Medicago truncatula] |  |
| Unigene0013502 | -2.54 | 0.008668785 | replication factor-A carboxy-terminal domain protein [Medicago truncatula] |  |
| Unigene0013681 | -3.07 | 0.002132433 | hypothetical protein MTR_1g080510 [Medicago truncatula] |  |
| Unigene0013687 | -5.29 | 0.045528272 | -- |  |
| Unigene0013788 | -1.66 | 0.044761345 | white-brown-complex ABC transporter family protein [Medicago truncatula] |  |
| Unigene0013841 | -12.17 | 1.46E-07 | PREDICTED: mediator-associated protein 1 [Cicer arietinum] |  |
| Unigene0013921 | -10.79 | 7.00E-05 | PREDICTED: ATP-dependent DNA helicase pfh1-like [Glycine max] |  |
| Unigene0013930 | -7.98 | 3.68E-06 | non-ltr retroelement reverse transcriptase [Rosa rugosa] |  |
| Unigene0013991 | -5.43 | 0.00127118 | -- |  |
| Unigene0014017 | -5.74 | 0.007595661 | DUF4283 domain protein [Medicago truncatula] |  |
| Unigene0014034 | -8.09 | 0.010273949 | PREDICTED: ATP-dependent DNA helicase PIF1-like [Erythranthe guttata] |  |
| Unigene0014046 | -8.87 | 1.87E-08 | Transposon Ty3-I Gag-Pol polyprotein [Cajanus cajan] |  |
| Unigene0014190 | -5.64 | 0.040701437 | -- |  |
| Unigene0014212 | -5.00 | 0.035421077 | -- |  |
| Unigene0014255 | -4.63 | 0.007249236 | SPX domain protein [Medicago truncatula] |  |
| Unigene0014404 | -6.94 | 0.02786969 | DNA replication factor CDT1-like protein [Medicago truncatula] |  |
| Unigene0014437 | -11.44 | 0.000787043 | -- |  |
| Unigene0014585 | -5.01 | 0.046745685 | PREDICTED: 9-cis-epoxycarotenoid dioxygenase NCED3, chloroplastic-like, partial [Cicer arietinum] |  |
| Unigene0015161 | -10.42 | 0.006985745 | ACT-like tyrosine kinase family protein [Medicago truncatula] |  |
| Unigene0015374 | -10.57 | 0.008827008 | RING-H2 zinc finger protein [Medicago truncatula] |  |
| Unigene0015550 | -11.39 | 0.002174916 | PREDICTED: pre-mRNA-splicing factor CWC22 homolog [Arachis ipaensis] |  |
| Unigene0015707 | -10.33 | 0.032449834 | DnaJ heat shock amino-terminal domain protein [Medicago truncatula] |  |
| Unigene0016011 | -10.41 | 0.039623255 | ribosomal RNA processing-like protein [Medicago truncatula] |  |
| Unigene0016200 | -10.90 | 3.05E-06 | PREDICTED: zinc finger BED domain-containing protein RICESLEEPER 2-like [Beta vulgaris subsp. vulgaris] [Beta vulgaris] |  |
| Unigene0016329 | -10.92 | 0.003573294 | Retrovirus-related Pol polyprotein from transposon TNT 1-94 [Cajanus cajan] |  |
| Unigene0016330 | -11.32 | 0.004015242 | -- |  |
| Unigene0016728 | -9.34 | 4.96E-06 | transmembrane amino acid transporter family protein [Medicago truncatula] |  |
| Unigene0016795 | -5.68 | 0.039623255 | FHA domain protein [Medicago truncatula] |  |
| Unigene0016820 | -11.79 | 0.043282983 | PREDICTED: uncharacterized protein LOC108193505 isoform X1 [Daucus carota subsp. sativus] [Daucus carota] |  |
| Unigene0017223 | -10.73 | 0.002890743 | Os01g0177800 [Oryza sativa Japonica Group] |  |
| Unigene0017830 | -5.09 | 0.017995618 | RALF [Medicago truncatula] |  |
| Unigene0017910 | -4.43 | 0.036916979 | -- |  |
| Unigene0017994 | -12.22 | 0.000185869 | -- |  |
| Unigene0018712 | -6.26 | 0.017745359 | LRR receptor-like kinase family protein [Medicago truncatula] |  |
| Unigene0018759 | -10.51 | 0.018687328 | cytochrome P450 family protein [Medicago truncatula] |  |
| Unigene0018988 | -11.42 | 0.000996378 | -- |  |
| Unigene0019019 | -13.13 | 1.09E-05 | -- |  |
| Unigene0019358 | -12.66 | 0.000893737 | -- |  |
| Unigene0019683 | -10.22 | 0.041555762 | serine kinase-like protein [Medicago truncatula] |  |
| Unigene0019943 | -5.07 | 0.034847703 | ubiquitin-protein ligase [Medicago truncatula] |  |
| Unigene0020077 | -3.42 | 0.022518841 | -- |  |
| Unigene0020135 | -3.16 | 0.003488863 | PREDICTED: probable DNA replication complex GINS protein PSF3 [Arachis ipaensis] |  |
| Unigene0020513 | -5.60 | 0.048561215 | ACT-like tyrosine kinase family protein [Medicago truncatula] |  |
| Unigene0020701 | -11.56 | 0.015194598 | -- |  |
| Unigene0021523 | -3.34 | 0.044965767 | expansin A10 [Medicago truncatula] |  |
| Unigene0021583 | -12.57 | 0.001499929 | E3 ubiquitin-protein ligase UPL6 [Cajanus cajan] |  |
| Unigene0021916 | -11.27 | 0.001798352 | hypothetical protein MTR_7g026040 [Medicago truncatula] |  |
| Unigene0022594 | -3.33 | 0.0489984 | -- |  |
| Unigene0022723 | -10.21 | 0.021247153 | animal RPA1 domain protein [Medicago truncatula] |  |
| Unigene0022784 | -10.29 | 0.046328551 | replication factor-A carboxy-terminal domain protein [Medicago truncatula] |  |
| Unigene0022942 | -12.89 | 0.014007558 | pre-rRNA-processing protein TSR2 [Medicago truncatula] |  |
| Unigene0023065 | -5.09 | 0.000117735 | RALF [Medicago truncatula] |  |
| Unigene0023387 | -4.48 | 0.031213513 | BnaC09g48580D [Brassica napus] |  |
| Unigene0023511 | -4.28 | 0.037423884 | -- |  |
| Unigene0023590 | -5.29 | 0.017990997 | -- |  |
| Unigene0023794 | -3.06 | 0.020500666 | homeobox associated leucine zipper protein [Medicago truncatula] |  |
| Unigene0024205 | -10.93 | 0.008640882 | PREDICTED: zinc finger protein 8 [Cicer arietinum] |  |
| Unigene0024235 | -10.90 | 0.003635433 | hypothetical protein MTR_0093s0130 [Medicago truncatula] |  |
| Unigene0024244 | -5.95 | 0.00443684 | -- |  |
| Unigene0024501 | -2.91 | 0.033425478 | thioredoxin [Medicago truncatula] |  |
| Unigene0024783 | -11.47 | 0.003243407 | -- |  |
| Unigene0024784 | -8.17 | 1.36E-06 | -- |  |
| Unigene0024984 | -3.63 | 0.02216115 | ovate transcriptional repressor [Medicago truncatula] |  |
| Unigene0024994 | -10.93 | 0.010993135 | sesquiterpene synthase [Medicago truncatula] |  |
| Unigene0025503 | -12.19 | 5.50E-05 | hypothetical protein MTR_5g081340 [Medicago truncatula] |  |
| Unigene0025882 | -2.64 | 0.036901243 | auxin-binding protein ABP19a [Medicago truncatula] |  |
| Unigene0025991 | -2.15 | 0.047893631 | DUF581 family protein [Medicago truncatula] |  |
| Unigene0026179 | -11.34 | 0.000132177 | kinase 1B [Medicago truncatula] |  |
| Unigene0026273 | -2.35 | 0.00098938 | BnaC05g08810D [Brassica napus] |  |
| Unigene0026642 | -5.39 | 0.024735161 | NB-ARC domain disease resistance protein [Medicago truncatula] |  |
| Unigene0026798 | -10.63 | 0.029436128 | -- |  |
| Unigene0027311 | -1.83 | 0.03887852 | ethylene receptor [Medicago truncatula] |  |
| Unigene0027439 | -2.42 | 0.010455751 | S-adenosyl-L-methionine:trans-caffeoyl-CoA 3-O-methyltransferase [Medicago sativa] |  |
| Unigene0027573 | -10.81 | 0.033425478 | hypothetical protein MTR_3g462090 [Medicago truncatula] |  |
| Unigene0027574 | -9.31 | 0.005852769 | hypothetical protein MTR_3g462080 [Medicago truncatula] |  |
| Unigene0027592 | -6.51 | 0.008640882 | Ovarian tumour, otubain [Medicago truncatula] |  |
| Unigene0028203 | -3.48 | 0.008278186 | feronia receptor-like kinase [Medicago truncatula] |  |
| Unigene0028385 | -1.85 | 0.004992584 | Cys2-His2 zinc finger transcription factor [Medicago truncatula] |  |
| Unigene0028494 | -7.31 | 0.000249885 | elongation factor 1-alpha [Medicago truncatula] |  |
| Unigene0028587 | -3.99 | 0.002611871 | DUF506 family protein [Medicago truncatula] |  |
| Unigene0028654 | -4.13 | 0.039322112 | PREDICTED: N6-adenosine-methyltransferase MT-A70-like [Vitis vinifera] |  |
| Unigene0028700 | -5.25 | 0.026892915 | cytokinin oxidase/dehydrogenase-like protein [Medicago truncatula] |  |
| Unigene0028930 | -12.00 | 2.58E-05 | PREDICTED: fasciclin-like arabinogalactan protein 21 [Arachis ipaensis] |  |
| Unigene0028962 | -5.99 | 0.024728867 | leguminosin proline-rich group669 secreted peptide [Medicago truncatula] |  |
| Unigene0029077 | -6.62 | 0.008816433 | -- |  |
| Unigene0029080 | -2.97 | 0.030270637 | Kunitz type trypsin inhibitor [Medicago truncatula] |  |
| Unigene0029152 | -2.91 | 0.002993739 | basic helix loop helix (bHLH) DNA-binding family protein [Medicago truncatula] |  |
| Unigene0029411 | -7.43 | 0.002134277 | cytochrome P450 family 82 protein [Medicago truncatula] |  |
| Unigene0029412 | -11.58 | 0.018261304 | PREDICTED: cytochrome P450 CYP82D47-like [Glycine max] |  |
| Unigene0029462 | -4.37 | 0.029767936 | receptor-like kinase feronia-like protein [Medicago truncatula] |  |
| Unigene0029791 | -3.08 | 0.043553026 | gibberellin 20-oxidase [Medicago truncatula] |  |
| Unigene0029796 | -2.08 | 0.021879615 | monothiol glutaredoxin-S2 protein [Medicago truncatula] |  |
| Unigene0029901 | -3.68 | 0.035073564 | -- |  |
| Unigene0030171 | -3.35 | 0.024216676 | PREDICTED: proteoglycan 4-like [Vigna angularis] |  |
| Unigene0030234 | -5.21 | 0.016598092 | expansin A10 [Medicago truncatula] |  |
| Unigene0030272 | -11.66 | 0.019264629 | -- |  |
| Unigene0030651 | -3.00 | 0.022004764 | basic helix loop helix (bHLH) family transcription factor [Medicago truncatula] |  |
| Unigene0030749 | -2.92 | 0.027941987 | PREDICTED: BRI1 kinase inhibitor 1-like [Cicer arietinum] |  |
| Unigene0030819 | -7.83 | 0.035421077 | PPR containing plant-like protein [Medicago truncatula] |  |
| Unigene0031168 | -3.69 | 0.013324973 | 1-deoxy-D-xylulose-5-phosphate synthase [Medicago truncatula] |  |
| Unigene0031463 | -10.65 | 0.009459852 | PREDICTED: traB domain-containing protein-like [Cicer arietinum] |  |
| Unigene0031800 | -11.71 | 1.97E-05 | -- |  |
| Unigene0032008 | -2.34 | 0.003889718 | RING-H2 zinc finger protein RHA2b [Cajanus cajan] |  |
| Unigene0032073 | -8.93 | 1.72E-08 | -- |  |
| Unigene0032176 | -10.31 | 0.04954846 | -- |  |
| Unigene0032232 | -2.13 | 0.003889718 | hypothetical protein MTR_5g009290 [Medicago truncatula] |  |
| Unigene0032442 | -10.59 | 0.004531815 | PREDICTED: replication protein A 70 kDa DNA-binding subunit B-like [Erythranthe guttata] |  |
| Unigene0032778 | -2.57 | 0.031069331 | PREDICTED: protein indeterminate-domain 9 [Cicer arietinum] |  |
| Unigene0032933 | -10.49 | 0.040701437 | -- |  |
| Unigene0033001 | -3.59 | 0.013443045 | homeobox leucine zipper ATHB-like protein [Medicago truncatula] |  |
| Unigene0033059 | -11.44 | 0.002556138 | disease resistance protein (TIR-NBS-LRR class) [Medicago truncatula] |  |
| Unigene0034099 | -8.38 | 0.000619904 | -- |  |
| Unigene0034327 | -2.94 | 0.043515904 | Ulp1 protease family, carboxy-terminal domain protein [Medicago truncatula] |  |
| Unigene0034385 | -2.88 | 0.0489984 | -- |  |
| Unigene0034591 | -3.03 | 0.018035282 | LRR receptor-like kinase family protein [Medicago truncatula] |  |
| Unigene0034601 | -2.08 | 0.007448122 | DUF4408 domain protein [Medicago truncatula] |  |
| Unigene0034756 | -12.05 | 0.012588637 | stress enhanced protein [Medicago truncatula] |  |
| Unigene0034849 | -2.79 | 0.033923814 | DUF223 domain protein [Medicago truncatula] |  |
| Unigene0035174 | -7.19 | 0.005549865 | transmembrane protein, putative [Medicago truncatula] |  |
| Unigene0035444 | -3.52 | 0.043445764 | -- |  |
| Unigene0035484 | -11.12 | 0.023959119 | -- |  |
| Unigene0035746 | -2.43 | 0.018261304 | cinnamyl alcohol dehydrogenase-like protein [Medicago truncatula] |  |
| Unigene0036309 | -3.14 | 0.023466584 | LRR receptor-like kinase [Medicago truncatula] |  |
| Unigene0036417 | -2.42 | 0.024336743 | RecName: Full=Early nodulin-12A; Short=N-12A; Flags: Precursor [Pisum sativum] |  |
| Unigene0036666 | -3.79 | 0.020515297 | feronia receptor-like kinase [Medicago truncatula] |  |
| Unigene0036708 | -10.95 | 0.016598092 | -- |  |
| Unigene0036871 | -11.35 | 0.010273949 | disease resistance protein (NBS-LRR class) family protein, partial [Medicago truncatula] |  |
| Unigene0036960 | -10.12 | 0.018815127 | PREDICTED: uncharacterized protein LOC101497185 [Cicer arietinum] |  |
| Unigene0037061 | -3.78 | 0.040686819 | receptor-like kinase [Medicago truncatula] |  |
| Unigene0037389 | -11.89 | 4.61E-05 | Tic22 family protein [Medicago truncatula] |  |
| Unigene0037394 | -12.70 | 0.034594289 | salicylic acid carboxyl methyltransferase [Medicago truncatula] |  |
| Unigene0037396 | -2.81 | 0.048720746 | 2-hydroxyisoflavanone dehydratase [Medicago truncatula] |  |
| Unigene0037398 | -1.92 | 0.016550132 | salicylic acid carboxyl methyltransferase [Medicago truncatula] |  |
| Unigene0037584 | -3.27 | 0.002473103 | Pmr5/Cas1p GDSL/SGNH-like acyl-esterase family protein [Medicago truncatula] |  |
| Unigene0037590 | -4.76 | 0.026746844 | -- |  |
| Unigene0037719 | -3.40 | 0.004348684 | transmembrane protein, putative [Medicago truncatula] |  |
| Unigene0037812 | -1.77 | 0.039953316 | beta-galactosidase [Medicago truncatula] |  |
| Unigene0037851 | -3.75 | 0.028836587 | disease resistance protein (TIR-NBS-LRR class) [Medicago truncatula] |  |
| Unigene0038102 | -11.20 | 0.019161661 | -- |  |
| Unigene0038208 | -10.80 | 0.009577835 | glycoside hydrolase family 18 protein [Medicago truncatula] |  |
| Unigene0038265 | -7.70 | 1.75E-10 | receptor-like kinase feronia-like protein [Medicago truncatula] |  |
| Unigene0038996 | -10.38 | 0.047485129 | LRR receptor-like kinase [Medicago truncatula] |  |
| Unigene0039251 | -10.78 | 0.021118475 | PREDICTED: vitellogenin-2-like [Arachis ipaensis] |  |
| Unigene0039268 | -12.19 | 0.001024461 | -- |  |
| Unigene0039476 | -2.90 | 0.026099661 | cytochrome P450 family monooxygenase [Medicago truncatula] |  |
| Unigene0039883 | -7.76 | 4.96E-06 | -- |  |
| Unigene0039929 | -2.23 | 0.011999868 | cysteine-rich receptor-kinase-like protein [Medicago truncatula] |  |
| Unigene0040290 | -3.77 | 0.03203935 | importin-like protein [Medicago truncatula] |  |
| Unigene0040291 | -10.97 | 0.006774791 | -- |  |
| Unigene0040468 | -1.72 | 0.00741945 | RING-H2 zinc finger protein [Medicago truncatula] |  |
| Unigene0040872 | -10.08 | 4.20E-11 | transmembrane protein, putative [Medicago truncatula] |  |
| Unigene0041123 | -3.64 | 0.009215584 | 60S ribosomal L8-like protein [Medicago truncatula] |  |
| Unigene0041171 | -8.51 | 0.01645805 | -- |  |
| Unigene0041192 | -3.31 | 0.040791389 | -- |  |
| Unigene0041251 | -2.05 | 0.016742002 | tyrosine kinase family protein [Medicago truncatula] |  |
| Unigene0041287 | -11.52 | 0.006328811 | -- |  |
| Unigene0041292 | -12.17 | 0.015278708 | -- |  |
| Unigene0041312 | -1.96 | 0.044761345 | glutathione S-transferase, amino-terminal domain protein [Medicago truncatula] |  |
| Unigene0041377 | -3.70 | 0.002993739 | hypothetical protein MTR_1342s0020 [Medicago truncatula] |  |
| Unigene0041416 | -5.18 | 0.01332279 | LRR receptor-like kinase family protein [Medicago truncatula] |  |
| Unigene0041568 | -5.97 | 0.001754158 | ankyrin repeat protein [Medicago truncatula] |  |
| Unigene0041604 | -3.48 | 0.010222982 | LRR receptor-like kinase family protein [Medicago truncatula] |  |
| Unigene0041700 | -2.60 | 0.005028099 | WRKY family transcription factor [Medicago truncatula] |  |
| Unigene0041801 | -6.87 | 0.029964637 | PREDICTED: plectin isoform X1 [Arachis duranensis] |  |
| Unigene0041809 | -1.58 | 0.031740732 | trihelix transcription factor GT-like protein [Medicago truncatula] |  |
| Unigene0041891 | -1.44 | 0.035612084 | LMBR1 integral membrane-like protein [Medicago truncatula] |  |
| Unigene0042141 | -10.15 | 0.026211841 | -- |  |
| Unigene0042225 | -12.91 | 0.028911899 | -- |  |
| Unigene0042529 | -11.51 | 0.025365709 | LRR receptor-like kinase family protein [Medicago truncatula] |  |
| Unigene0042636 | -2.94 | 0.043282983 | albumin-2 protein [Medicago truncatula] |  |
| Unigene0042652 | -11.44 | 0.009189382 | expansin A1 [Medicago truncatula] |  |
| Unigene0042749 | -1.79 | 0.011081909 | beta-like galactosidase [Medicago truncatula] |  |
| Unigene0042760 | -4.68 | 0.000499497 | -- |  |
| Unigene0042841 | -3.57 | 0.000417402 | ethylene-responsive transcription factor ERF017-like protein [Medicago truncatula] |  |
| Unigene0043164 | -10.75 | 0.007184016 | -- |  |
| Unigene0043319 | -11.08 | 0.009388502 | hypothetical protein MTR_3g027045 [Medicago truncatula] |  |
| Unigene0043599 | -4.11 | 0.016598092 | flavonoid glucosyltransferase [Medicago truncatula] |  |
| Unigene0043644 | -11.32 | 3.96E-05 | -- |  |
| Unigene0043844 | -4.42 | 0.047893631 | hypothetical protein MTR_4g007150 [Medicago truncatula] |  |
| Unigene0043949 | -9.95 | 0.027073428 | -- |  |
| Unigene0044027 | -5.83 | 0.00098938 | -- |  |
| Unigene0044155 | -14.22 | 1.80E-10 | -- |  |
| Unigene0044262 | -3.07 | 1.21E-05 | auxin efflux carrier family transporter [Medicago truncatula] |  |
| Unigene0044370 | -3.81 | 0.001880743 | hypothetical protein MTR_5g098775 [Medicago truncatula] |  |
| Unigene0044746 | -10.70 | 0.006200948 | auxin response factor 14 [Medicago truncatula] |  |
| Unigene0044999 | -1.77 | 0.022050176 | ARM repeat CCCH-type zinc finger protein [Medicago truncatula] |  |
| Unigene0045069 | -5.32 | 0.007620159 | hypothetical protein MTR_4g098970 [Medicago truncatula] |  |
| Unigene0045128 | -11.68 | 0.011999868 | COP9 signalosome complex subunit-like protein [Medicago truncatula] |  |
| Unigene0045172 | -10.65 | 0.006527318 | UDP-glucosyltransferase family protein [Medicago truncatula] |  |
| Unigene0045203 | -5.63 | 0.01256078 | pectinesterase/pectinesterase inhibitor [Medicago truncatula] |  |
| Unigene0045492 | -5.02 | 0.049800301 | -- |  |
| Unigene0045503 | -3.21 | 0.004862286 | (3S)-linalool/(E)-nerolidol/(E,E)-geranyl linalool synthase [Medicago truncatula] |  |
| Unigene0045561 | -5.19 | 0.001935815 | receptor-like protein [Medicago truncatula] |  |
| Unigene0045589 | -10.00 | 5.36E-09 | copine protein [Medicago truncatula] |  |
| Unigene0045636 | -11.03 | 0.007691708 | -- |  |
| Unigene0045810 | -11.31 | 0.015671833 | disease resistance protein (TIR-NBS-LRR class) [Medicago truncatula] |  |
| Unigene0046126 | -3.74 | 0.000967372 | 1-deoxy-D-xylulose-5-phosphate synthase [Medicago truncatula] |  |
| Unigene0046171 | -7.95 | 1.69E-06 | Retrovirus-related Pol polyprotein from transposon TNT 1-94, partial [Glycine soja] |  |
| Unigene0046284 | -2.27 | 0.035073564 | homeobox leucine zipper family protein [Medicago truncatula] |  |
| Unigene0046531 | -4.11 | 0.014506297 | PREDICTED: E3 ubiquitin-protein ligase UPL6 [Cicer arietinum] |  |
| Unigene0046534 | -10.05 | 0.01596238 | E3 ubiquitin-protein ligase UPL6 [Medicago truncatula] |  |
| Unigene0046551 | -12.71 | 1.08E-07 | PREDICTED: pre-rRNA-processing protein TSR2 homolog [Ziziphus jujuba] |  |
| Unigene0046574 | -4.55 | 0.013486377 | -- |  |
| Unigene0046714 | -11.05 | 0.007704696 | Pmr5/Cas1p GDSL/SGNH-like acyl-esterase family protein [Medicago truncatula] |  |
| Unigene0046721 | -9.88 | 4.96E-06 | -- |  |
| Unigene0046882 | -2.26 | 0.008371123 | F-box plant-like protein [Medicago truncatula] |  |
| Unigene0047038 | -5.81 | 0.003488863 | frigida interacting protein [Medicago truncatula] |  |
| Unigene0047190 | -10.38 | 0.031597011 | -- |  |
| Unigene0047244 | -2.57 | 7.19E-05 | tryptophan synthase beta type 2 [Medicago truncatula] |  |
| Unigene0047452 | -5.03 | 2.54E-06 | PREDICTED: chitin elicitor receptor kinase 1-like isoform X2 [Cicer arietinum] |  |
| Unigene0047526 | -10.60 | 0.021828656 | PREDICTED: transcription factor DIVARICATA-like [Cicer arietinum] |  |
| Unigene0047766 | -2.88 | 0.045528272 | LRR receptor-like kinase family protein [Medicago truncatula] |  |
| Unigene0047910 | -6.42 | 0.001880743 | transmembrane protein, putative [Medicago truncatula] |  |
| Unigene0047980 | -2.07 | 0.011929214 | carboxylesterase-like protein [Medicago truncatula] |  |
| Unigene0048057 | -2.18 | 0.025521715 | calcium-binding EF-hand protein [Medicago truncatula] |  |
| Unigene0048131 | -2.42 | 0.013324709 | ethylene-responsive transcription factor ERF017-like protein [Medicago truncatula] |  |
| Unigene0048365 | -1.97 | 0.048710087 | homeobox leucine zipper protein [Medicago truncatula] |  |
| Unigene0048737 | -2.36 | 0.027041756 | S-locus lectin kinase family protein [Medicago truncatula] |  |
| Unigene0048743 | -3.79 | 0.025171254 | hypothetical protein MTR_8g070580 [Medicago truncatula] |  |
| Unigene0048812 | -7.12 | 0.011409804 | replication factor-A carboxy-terminal domain protein [Medicago truncatula] |  |
| Unigene0048949 | -3.98 | 0.01596238 | NB-ARC domain disease resistance protein [Medicago truncatula] |  |
| Unigene0049383 | -6.61 | 0.019901177 | ubiquitin ATG12-like protein [Medicago truncatula] |  |
| Unigene0049746 | -3.19 | 0.045921376 | LRR receptor-like kinase [Medicago truncatula] |  |
| Unigene0049824 | -5.64 | 4.99E-05 | (E)-beta-ocimene/myrcene synthase [Medicago truncatula] |  |
| Unigene0049877 | -1.82 | 0.036697416 | DUF4228 domain protein [Medicago truncatula] |  |
| Unigene0049892 | -2.00 | 0.041414718 | seed linoleate 9S-lipoxygenase [Medicago truncatula] |  |
| Unigene0050192 | -3.26 | 0.003480505 | hypothetical protein MTR_6g052610 [Medicago truncatula] |  |
| Unigene0050595 | -2.60 | 0.034445407 | L-type lectin-domain receptor kinase S.4 [Medicago truncatula] |  |
| Unigene0050632 | -1.70 | 0.02812805 | homeobox leucine zipper protein [Medicago truncatula] |  |
| Unigene0050714 | -10.85 | 0.023761985 | Transposon Ty3-G Gag-Pol polyprotein [Cajanus cajan] |  |
| Unigene0050758 | -1.62 | 0.036916979 | BEL1-related homeotic protein [Medicago truncatula] |  |
| Unigene0050873 | -5.25 | 0.039953316 | LRR receptor-like kinase [Medicago truncatula] |  |
| Unigene0050983 | -5.75 | 0.002568075 | -- |  |
| Unigene0051005 | -3.23 | 0.005018405 | peptide transporter [Medicago truncatula] |  |
| Unigene0051168 | -14.28 | 1.03E-11 | -- |  |
| Unigene0051269 | -12.66 | 3.84E-05 | -- |  |
| Unigene0051347 | -11.14 | 0.021480609 | carbohydrate-binding protein of the ER protein [Medicago truncatula] |  |
| Unigene0051383 | -4.36 | 0.04308217 | replication factor-A carboxy-terminal domain protein [Medicago truncatula] |  |
| Unigene0051601 | -2.60 | 0.003488863 | hypothetical protein MTR_4g016760 [Medicago truncatula] |  |
| Unigene0051606 | -14.59 | 8.19E-09 | -- |  |
| Unigene0051788 | -3.35 | 0.039532714 | Ulp1 protease family, carboxy-terminal domain protein [Medicago truncatula] |  |
| Unigene0052089 | -13.64 | 2.69E-06 | -- |  |
| Unigene0052439 | -9.95 | 2.34E-08 | Retrovirus-related Pol polyprotein LINE-1, partial [Cajanus cajan] |  |
| Unigene0052443 | -4.33 | 0.026746844 | G-type lectin S-receptor-like Serine/Threonine-kinase [Medicago truncatula] |  |
| Unigene0052500 | -11.17 | 0.037494188 | PREDICTED: serine/threonine-protein kinase Aurora-2 [Nicotiana sylvestris] |  |
| Unigene0053032 | -1.95 | 0.045739518 | cryptochrome 2B apoprotein [Medicago truncatula] |  |
| Unigene0053068 | -10.96 | 0.048288328 | -- |  |
| Unigene0053251 | -1.77 | 0.041837285 | phytochrome B, partial [Medicago sativa] |  |
| Unigene0053432 | -2.53 | 0.004263413 | auxin response factor-like protein [Medicago truncatula] |  |
| Unigene0053465 | -5.23 | 0.014597675 | hypothetical protein MTR_5g045335 [Medicago truncatula] |  |
| Unigene0053548 | -11.86 | 0.001127994 | -- |  |
| Unigene0053652 | -9.28 | 0.01256078 | -- |  |
| Unigene0053820 | -11.31 | 0.024735161 | senescence-associated protein [Medicago truncatula] |  |
| Unigene0053870 | -2.27 | 0.003126452 | ankyrin repeat plant-like protein [Medicago truncatula] |  |
| Unigene0053873 | -2.05 | 0.013751415 | PREDICTED: 1-aminocyclopropane-1-carboxylate oxidase homolog 1-like [Cicer arietinum] |  |
| Unigene0054125 | -5.21 | 0.00489859 | disease resistance protein (TIR-NBS-LRR class), partial [Medicago truncatula] |  |
| Unigene0055693 | -5.40 | 0.0479541 | -- |  |
| Unigene0055743 | -6.52 | 0.047435168 | cyclic nucleotide-gated cation channel protein [Medicago truncatula] |  |
| Unigene0056088 | -3.05 | 0.007249236 | receptor-like kinase feronia-like protein [Medicago truncatula] |  |
| Unigene0056475 | -10.39 | 0.008898138 | replication factor-A carboxy-terminal domain protein [Medicago truncatula] |  |
| Unigene0056814 | -12.15 | 1.74E-06 | transmembrane protein, putative [Medicago truncatula] |  |
| Unigene0057193 | -6.35 | 0.001741913 | WRKY transcription factor [Medicago truncatula] |  |
| Unigene0057205 | -6.62 | 0.015616352 | hypothetical protein MTR_8g051830 [Medicago truncatula] |  |
| Unigene0057337 | -10.85 | 0.042782194 | -- |  |
| Unigene0057543 | -11.74 | 0.014822157 | Polynucleotidyl transferase, Ribonuclease H fold [Medicago truncatula] |  |
| Unigene0057905 | -6.81 | 0.003939693 | Nucleic acid-binding, OB-fold [Medicago truncatula] |  |
| Unigene0059248 | -11.28 | 0.000203333 | Serine/threonine protein phosphatase 7 long form isogeny, partial [Cajanus cajan] |  |
| Unigene0059635 | -12.04 | 0.00245259 | 40S ribosomal protein S24-2 [Medicago truncatula] |  |
| Unigene0060076 | -11.97 | 8.17E-10 | DUF4283 domain protein [Medicago truncatula] |  |
| Unigene0060258 | -11.08 | 0.026740459 | hypothetical protein MTR_0202s0040 [Medicago truncatula] |  |
| Unigene0060283 | -4.54 | 0.012172516 | RALF [Medicago truncatula] |  |
| Unigene0060508 | -11.13 | 0.012588637 | -- |  |
| Unigene0060974 | -7.85 | 0.044435971 | ATP-dependent chaperone ClpB [Medicago truncatula] |  |
| Unigene0061472 | -10.87 | 0.014854974 | Polynucleotidyl transferase, Ribonuclease H fold [Medicago truncatula] |  |
| Unigene0061630 | -11.09 | 0.032873729 | disease resistance protein (NBS-LRR class) family protein [Medicago truncatula] |  |
| Unigene0061832 | -10.48 | 0.047893631 | -- |  |
| Unigene0062427 | -11.66 | 0.012677232 | ATP-dependent chaperone ClpB [Medicago truncatula] |  |
| Unigene0062434 | -6.05 | 0.020121576 | Polynucleotidyl transferase, Ribonuclease H fold [Medicago truncatula] |  |
| Unigene0062731 | -9.88 | 0.020157713 | hypothetical protein MTR_5g042630 [Medicago truncatula] |  |
| Unigene0062758 | -11.09 | 0.017125547 | Polynucleotidyl transferase, Ribonuclease H fold [Medicago truncatula] |  |
| Unigene0063070 | -10.69 | 0.024735161 | -- |  |
| Unigene0063087 | -10.26 | 0.037272217 | -- |  |
| Unigene0063257 | -10.62 | 0.024641513 | PREDICTED: TMV resistance protein N-like isoform X1 [Cicer arietinum] |  |
| Unigene0063299 | -10.63 | 0.011959552 | -- |  |
| Unigene0063563 | -9.88 | 0.045953659 | plastid transcriptionally active protein [Medicago truncatula] |  |
| Unigene0064562 | -11.68 | 0.003243407 | hypothetical protein MTR_7g067950 [Medicago truncatula] |  |
| Unigene0064922 | -10.68 | 0.01542643 | Copia protein [Cajanus cajan] |  |
| Unigene0065062 | -10.89 | 0.013094783 | PREDICTED: mitogen-activated protein kinase kinase kinase 1-like isoform X2 [Glycine max] |  |
| Unigene0065233 | -11.93 | 0.026746844 | unknown [Medicago truncatula] |  |
| Unigene0065234 | -11.20 | 0.007249236 | -- |  |
| Unigene0065290 | -11.92 | 1.84E-05 | hypothetical protein MTR_3g063060 [Medicago truncatula] |  |
| Unigene0065346 | -6.61 | 0.04954846 | -- |  |
| Unigene0066377 | -9.71 | 8.17E-10 | C2H2-type zinc finger protein [Medicago truncatula] |  |
| Unigene0066389 | -10.34 | 0.044052956 | -- |  |
| Unigene0066471 | -12.66 | 1.45E-05 | PREDICTED: ABA-responsive protein ABR17-like [Vigna radiata var. radiata] [Vigna radiata] |  |
| Unigene0066472 | -11.75 | 0.012456554 | -- |  |
| Unigene0066503 | -10.61 | 0.042907713 | -- |  |
| Unigene0066619 | -10.41 | 0.021296957 | F-box/LRR protein [Medicago truncatula] |  |
