# Supplementary material for: Study on differentially expressed genes related to defoliation traits in two alfalfa varieties based on RNA-Seq
Source: BMC Genomics. 2018 Nov 7;19:807. doi: 10.1186/s12864-018-5180-1 (PMC6223052; doi:10.1186/s12864-018-5180-1)
Supplement: Supplementary file 2 — Table S2. GO functional annotations and the number of DEGs statistics. (DOCX 18 kb) [file 12864_2018_5180_MOESM2_ESM.docx]

| Ontology | Class | GO ID | No. of up-regulation unigenes | No. of down-regulation unigenes | Total |
| --- | --- | --- | --- | --- | --- |
| Biological Process | biological regulation | 0065007 | 25 | 12 | 37 |
|  | cellular component organization or biogenesis | 0071840 | 45 | 14 | 59 |
|  | cellular process | 0009987 | 170 | 90 | 260 |
|  | developmental process | 0032502 | 23 | 14 | 37 |
|  | growth | 0040007 | 3 | 2 | 5 |
|  | immune system process | 0002376 | 1 | 1 | 2 |
|  | localization | 0051179 | 21 | 9 | 30 |
|  | metabolic process | 0008152 | 195 | 93 | 288 |
|  | multi-organism process | 0051704 | 21 | 8 | 29 |
|  | multicellular organismal process | 0032501 | 18 | 6 | 24 |
|  | reproduction | 0000003 | 6 | 5 | 11 |
|  | reproductive process | 0022414 | 6 | 5 | 11 |
|  | response to stimulus | 0050896 | 131 | 43 | 174 |
|  | rhythmic process | 0048511 | 0 | 2 | 2 |
|  | signaling | 0023052 | 11 | 5 | 16 |
|  | single-organism process | 0044699 | 109 | 50 | 159 |
| Cellular Component | cell | 0005623 | 210 | 46 | 256 |
|  | cell junction | 0030054 | 39 | 4 | 43 |
|  | cell part | 0044464 | 210 | 46 | 256 |
|  | extracellular region | 0005576 | 7 | 2 | 9 |
|  | macromolecular complex | 0032991 | 88 | 3 | 91 |
|  | membrane | 0016020 | 71 | 16 | 87 |
|  | membrane part | 0044425 | 14 | 8 | 22 |
|  | membrane-enclosed lumen | 0031974 | 38 | 4 | 42 |
|  | organelle | 0043226 | 156 | 30 | 186 |
|  | organelle part | 0044422 | 100 | 10 | 110 |
|  | supramolecular fiber | 0099512 | 1 | 0 | 1 |
| Molecular Function | antioxidant activity | 0016209 | 1 | 1 | 2 |
|  | binding | 0005488 | 169 | 97 | 266 |
|  | catalytic activity | 0003824 | 124 | 99 | 223 |
|  | molecular function regulator | 0098772 | 4 | 3 | 7 |
|  | molecular transducer activity | 0060089 | 1 | 2 | 3 |
|  | nucleic acid binding transcription factor activity | 0001071 | 3 | 10 | 13 |
|  | signal transducer activity | 0004871 | 0 | 1 | 1 |
|  | structural molecule activity | 0005198 | 44 | 1 | 45 |
|  | transcription factor activity, protein binding | 0000988 | 1 | 0 | 1 |
|  | transporter activity | 0005215 | 11 | 3 | 14 |
