# Supplementary material for: Study on differentially expressed genes related to defoliation traits in two alfalfa varieties based on RNA-Seq
Source: BMC Genomics. 2018 Nov 7;19:807. doi: 10.1186/s12864-018-5180-1 (PMC6223052; doi:10.1186/s12864-018-5180-1)
Supplement: Supplementary file 3 — Table S3. KEGG pathway annotation of DEGs between two varieties of alfalfa leaves. (DOCX 26 kb) [file 12864_2018_5180_MOESM3_ESM.docx]

| **Pathway** | **All genes with pathway annotation (8414)** | **DEGs genes with pathway annotation (281)** | **Pvalue** | **Qvalue** | **Pathway ID** |
| --- | --- | --- | --- | --- | --- |
| Ribosome | 622 (7.39%) | 64 (22.78%) | 0 | 0 | ko03010 |
| Spliceosome | 562 (6.68%) | 19 (6.76%) | 0.512936 | 0.986478 | ko03040 |
| Protein processing in endoplasmic reticulum | 550 (6.54%) | 44 (15.66%) | 0 | 0.000002 | ko04141 |
| Carbon metabolism | 484 (5.75%) | 22 (7.83%) | 0.086372 | 0.903225 | ko01200 |
| Endocytosis | 442 (5.25%) | 9 (3.2%) | 0.9638 | 0.986478 | ko04144 |
| Biosynthesis of amino acids | 436 (5.18%) | 17 (6.05%) | 0.288391 | 0.986478 | ko01230 |
| RNA transport | 384 (4.56%) | 10 (3.56%) | 0.832935 | 0.986478 | ko03013 |
| Plant-pathogen interaction | 377 (4.48%) | 12 (4.27%) | 0.610856 | 0.986478 | ko04626 |
| Starch and sucrose metabolism | 329 (3.91%) | 8 (2.85%) | 0.86544 | 0.986478 | ko00500 |
| Oxidative phosphorylation | 304 (3.61%) | 12 (4.27%) | 0.317095 | 0.986478 | ko00190 |
| Nucleotide excision repair | 299 (3.55%) | 11 (3.91%) | 0.414994 | 0.986478 | ko03420 |
| Plant hormone signal transduction | 294 (3.49%) | 6 (2.14%) | 0.932493 | 0.986478 | ko04075 |
| DNA replication | 280 (3.33%) | 11 (3.91%) | 0.333529 | 0.986478 | ko03030 |
| Ubiquitin mediated proteolysis | 280 (3.33%) | 7 (2.49%) | 0.832451 | 0.986478 | ko04120 |
| Homologous recombination | 270 (3.21%) | 11 (3.91%) | 0.29201 | 0.986478 | ko03440 |
| Mismatch repair | 244 (2.9%) | 11 (3.91%) | 0.193 | 0.986478 | ko03430 |
| Pyrimidine metabolism | 240 (2.85%) | 1 (0.36%) | 0.999745 | 0.999745 | ko00240 |
| Phenylpropanoid biosynthesis | 226 (2.69%) | 4 (1.42%) | 0.947796 | 0.986478 | ko00940 |
| Amino sugar and nucleotide sugar metabolism | 210 (2.5%) | 5 (1.78%) | 0.836249 | 0.986478 | ko00520 |
| Glycolysis / Gluconeogenesis | 204 (2.42%) | 10 (3.56%) | 0.144849 | 0.986478 | ko00010 |
| Ribosome biogenesis in eukaryotes | 196 (2.33%) | 3 (1.07%) | 0.962293 | 0.986478 | ko03008 |
| RNA degradation | 195 (2.32%) | 4 (1.42%) | 0.895787 | 0.986478 | ko03018 |
| mRNA surveillance pathway | 192 (2.28%) | 3 (1.07%) | 0.958211 | 0.986478 | ko03015 |
| Pyruvate metabolism | 184 (2.19%) | 8 (2.85%) | 0.272724 | 0.986478 | ko00620 |
| Cysteine and methionine metabolism | 168 (2%) | 6 (2.14%) | 0.492706 | 0.986478 | ko00270 |
| Phagosome | 162 (1.93%) | 5 (1.78%) | 0.63433 | 0.986478 | ko04145 |
| Glutathione metabolism | 162 (1.93%) | 4 (1.42%) | 0.795476 | 0.986478 | ko00480 |
| Fatty acid metabolism | 158 (1.88%) | 5 (1.78%) | 0.612207 | 0.986478 | ko01212 |
| Peroxisome | 144 (1.71%) | 2 (0.71%) | 0.956353 | 0.986478 | ko04146 |
| Carbon fixation in photosynthetic organisms | 139 (1.65%) | 8 (2.85%) | 0.093437 | 0.903225 | ko00710 |
| Glyoxylate and dicarboxylate metabolism | 131 (1.56%) | 5 (1.78%) | 0.445751 | 0.986478 | ko00630 |
| Proteasome | 130 (1.55%) | 5 (1.78%) | 0.439164 | 0.986478 | ko03050 |
| Glycerophospholipid metabolism | 128 (1.52%) | 3 (1.07%) | 0.806541 | 0.986478 | ko00564 |
| 2-Oxocarboxylic acid metabolism | 120 (1.43%) | 6 (2.14%) | 0.212213 | 0.986478 | ko01210 |
| Citrate cycle (TCA cycle) | 119 (1.41%) | 12 (4.27%) | 0.000596 | 0.017283 | ko00020 |
| Galactose metabolism | 113 (1.34%) | 2 (0.71%) | 0.895999 | 0.986478 | ko00052 |
| Glycine, serine and threonine metabolism | 113 (1.34%) | 1 (0.36%) | 0.979027 | 0.990411 | ko00260 |
| Alanine, aspartate and glutamate metabolism | 109 (1.3%) | 2 (0.71%) | 0.884063 | 0.986478 | ko00250 |
| Pentose phosphate pathway | 102 (1.21%) | 7 (2.49%) | 0.05404 | 0.783574 | ko00030 |
| Fructose and mannose metabolism | 97 (1.15%) | 6 (2.14%) | 0.105248 | 0.915661 | ko00051 |
| Glycerolipid metabolism | 95 (1.13%) | 2 (0.71%) | 0.831688 | 0.986478 | ko00561 |
| Base excision repair | 93 (1.11%) | 1 (0.36%) | 0.958275 | 0.986478 | ko03410 |
| Pentose and glucuronate interconversions | 93 (1.11%) | 4 (1.42%) | 0.376953 | 0.986478 | ko00040 |
| Valine, leucine and isoleucine degradation | 93 (1.11%) | 2 (0.71%) | 0.822668 | 0.986478 | ko00280 |
| alpha-Linolenic acid metabolism | 93 (1.11%) | 4 (1.42%) | 0.376953 | 0.986478 | ko00592 |
| Fatty acid degradation | 92 (1.09%) | 3 (1.07%) | 0.598109 | 0.986478 | ko00071 |
| Cyanoamino acid metabolism | 89 (1.06%) | 2 (0.71%) | 0.80332 | 0.986478 | ko00460 |
| Phosphatidylinositol signaling system | 89 (1.06%) | 1 (0.36%) | 0.95213 | 0.986478 | ko04070 |
| Protein export | 89 (1.06%) | 1 (0.36%) | 0.95213 | 0.986478 | ko03060 |
| Biosynthesis of unsaturated fatty acids | 87 (1.03%) | 4 (1.42%) | 0.331072 | 0.986478 | ko01040 |
| Phenylalanine metabolism | 81 (0.96%) | 1 (0.36%) | 0.937007 | 0.986478 | ko00360 |
| Inositol phosphate metabolism | 79 (0.94%) | 1 (0.36%) | 0.932534 | 0.986478 | ko00562 |
| Tryptophan metabolism | 74 (0.88%) | 1 (0.36%) | 0.91992 | 0.986478 | ko00380 |
| Phenylalanine, tyrosine and tryptophan biosynthesis | 73 (0.87%) | 1 (0.36%) | 0.917128 | 0.986478 | ko00400 |
| Regulation of autophagy | 71 (0.84%) | 2 (0.71%) | 0.691647 | 0.986478 | ko04140 |
| Fatty acid biosynthesis | 71 (0.84%) | 1 (0.36%) | 0.91125 | 0.986478 | ko00061 |
| Terpenoid backbone biosynthesis | 69 (0.82%) | 3 (1.07%) | 0.40678 | 0.986478 | ko00900 |
| Basal transcription factors | 68 (0.81%) | 2 (0.71%) | 0.668646 | 0.986478 | ko03022 |
| Lysine degradation | 68 (0.81%) | 1 (0.36%) | 0.901645 | 0.986478 | ko00310 |
| Arginine biosynthesis | 68 (0.81%) | 1 (0.36%) | 0.901645 | 0.986478 | ko00220 |
| Propanoate metabolism | 67 (0.8%) | 3 (1.07%) | 0.38863 | 0.986478 | ko00640 |
| Circadian rhythm - plant | 61 (0.72%) | 4 (1.42%) | 0.145934 | 0.986478 | ko04712 |
| Ubiquinone and other terpenoid-quinone biosynthesis | 60 (0.71%) | 1 (0.36%) | 0.870663 | 0.986478 | ko00130 |
| Ascorbate and aldarate metabolism | 59 (0.7%) | 1 (0.36%) | 0.866161 | 0.986478 | ko00053 |
| Flavonoid biosynthesis | 54 (0.64%) | 1 (0.36%) | 0.841203 | 0.986478 | ko00941 |
| Tyrosine metabolism | 51 (0.61%) | 1 (0.36%) | 0.824054 | 0.986478 | ko00350 |
| Nitrogen metabolism | 48 (0.57%) | 1 (0.36%) | 0.805062 | 0.986478 | ko00910 |
| Butanoate metabolism | 45 (0.53%) | 1 (0.36%) | 0.784027 | 0.986478 | ko00650 |
| Carotenoid biosynthesis | 44 (0.52%) | 1 (0.36%) | 0.776524 | 0.986478 | ko00906 |
| Steroid biosynthesis | 44 (0.52%) | 1 (0.36%) | 0.776524 | 0.986478 | ko00100 |
| Pantothenate and CoA biosynthesis | 41 (0.49%) | 1 (0.36%) | 0.752422 | 0.986478 | ko00770 |
| Selenocompound metabolism | 39 (0.46%) | 1 (0.36%) | 0.734932 | 0.986478 | ko00450 |
| Valine, leucine and isoleucine biosynthesis | 36 (0.43%) | 4 (1.42%) | 0.030994 | 0.5393 | ko00290 |
| Linoleic acid metabolism | 32 (0.38%) | 1 (0.36%) | 0.663444 | 0.986478 | ko00591 |
| Sesquiterpenoid and triterpenoid biosynthesis | 30 (0.36%) | 2 (0.71%) | 0.264896 | 0.986478 | ko00909 |
| Monoterpenoid biosynthesis | 30 (0.36%) | 2 (0.71%) | 0.264896 | 0.986478 | ko00902 |
| Ether lipid metabolism | 29 (0.34%) | 1 (0.36%) | 0.627202 | 0.986478 | ko00565 |
| Diterpenoid biosynthesis | 29 (0.34%) | 3 (1.07%) | 0.071118 | 0.8839 | ko00904 |
| Arachidonic acid metabolism | 26 (0.31%) | 1 (0.36%) | 0.587072 | 0.986478 | ko00590 |
| Vitamin B6 metabolism | 25 (0.3%) | 2 (0.71%) | 0.202635 | 0.986478 | ko00750 |
| Stilbenoid, diarylheptanoid and gingerol biosynthesis | 23 (0.27%) | 1 (0.36%) | 0.54264 | 0.986478 | ko00945 |
| Thiamine metabolism | 21 (0.25%) | 4 (1.42%) | 0.00465 | 0.101132 | ko00730 |
| Biotin metabolism | 21 (0.25%) | 1 (0.36%) | 0.510403 | 0.986478 | ko00780 |
| C5-Branched dibasic acid metabolism | 17 (0.2%) | 1 (0.36%) | 0.438981 | 0.986478 | ko00660 |
| Zeatin biosynthesis | 16 (0.19%) | 1 (0.36%) | 0.419559 | 0.986478 | ko00908 |
| Synthesis and degradation of ketone bodies | 15 (0.18%) | 1 (0.36%) | 0.399467 | 0.986478 | ko00072 |
| Glucosinolate biosynthesis | 10 (0.12%) | 1 (0.36%) | 0.288127 | 0.986478 | ko00966 |
