# Supplementary material for: Study on differentially expressed genes related to defoliation traits in two alfalfa varieties based on RNA-Seq
Source: BMC Genomics. 2018 Nov 7;19:807. doi: 10.1186/s12864-018-5180-1 (PMC6223052; doi:10.1186/s12864-018-5180-1)
Supplement: Supplementary file 4 — Table S4. Genes for RT-qPCR analysis and the list of GO ID. (DOCX 14 kb) [file 12864_2018_5180_MOESM4_ESM.docx]

| **GeneID** | **Gene Name** | **GO ID** |
| --- | --- | --- |
| Unigene0044746 | Auxin response factor (*ARF*) | GO:0005634; GO:0003677; GO:0007165; GO:0034641; GO:0009058 |
| Unigene0002039 | Phytochrome interacting factor 3 (PIF3) | GO:0005515; GO:0005488 |
| Unigene0027311 | Ethylene receptor protein (ETR) | GO:0060089; GO:0006796; GO:0004673; GO:0007165; GO:0072328; GO:0032550 |
| Unigene0053251 | Phytochrome B (PHYB) | GO:0004673; GO:0032550 ; GO:0007602; GO:0009645; GO:0006833; GO:0006351; GO:0009755; GO:0006714; GO:0051276; GO:0006796; GO:0046983; GO:0009883; GO:0009845; GO:0009606 ; GO:0010017; GO:0006464; GO:0044451; GO:0006952; GO:0044444; GO:0007623 |
| Unigene0053032 | Cryptochrome (CRY) | GO:0046983; GO:0005515; GO:0016829; GO:0016830; GO:0005488; GO:0003824 |
| Unigene0014585 | 9-cis-epoxycarotenoid dioxygenase 3 (NCED3) | GO:0006461; GO:0006950; GO:0008152; GO:0032502; GO:0044710; GO:0022414; GO:0000003; GO:0003006; GO:0044699 |
| Unigene0030464 | Tubulin alpha (TUBA) | GO:0005856; GO:0007010; GO:0005737; GO:0003924; GO:0000902; GO:0005198; GO:0043167; GO:0030154; GO:0000003; GO:0040007 |
| Unigene0032887 | Indole-3-acetic acid-amido synthetase (GH) | GO:0042221; GO:0050896; GO:0010033; GO:0009719; GO:0009725 |
