# Supplementary material for: Study on differentially expressed genes related to defoliation traits in two alfalfa varieties based on RNA-Seq
Source: BMC Genomics. 2018 Nov 7;19:807. doi: 10.1186/s12864-018-5180-1 (PMC6223052; doi:10.1186/s12864-018-5180-1)
Supplement: Supplementary file 5 — Table S5. Primers used for RT-qPCR analysis. (DOCX 12 kb) [file 12864_2018_5180_MOESM5_ESM.docx]

| **Primers** | **Primer sequence** |
| --- | --- |
| 0030464 F | TTCGCCCGTGGTCATTATAC |
| 0030464 R | AAGAGAACCCAAACCAGATCC |
| 0032887 F | CTGACGACGGTCCATTTCTT |
| 0032887 R | GATCTTGTCCTCTCATCCAATCTC |
| 0002039 F | GCACCTAGAATTAGAGGAAAGAGAA |
| 0002039 R | TGAGGCTTCAAGTGAGCATAC |
| 0014585 F | GGTGGTTCTCCGGTGATATTC |
| 0014585 R | TCTGGTGATTCCACCCAAATAA |
| 0027311 F | GAAAGGGCAACTGCTACCT |
| 0027311 R | GGTTCTTATTCTCCCTACTGATAGTG |
| 0044746 F | TGGAATGCCCTTCAGCATAA |
| 0044746 R | CCTGGTTCAGAATGGAGATGTAT |
| 0053032 F | CAAGTCCAGCTCCAAGAGATT |
| 0053032 R | TTTGGTGTCCTAAAGAGGAAGG |
| 0053251 F | AAACGAGTCTCGCAGGATAAG |
| 0053251 R | TAAGGATGATGGGCAGAGAATG |
| Ms GAPDH F | CCCTCTCCCTGTACAAAACTC |
| Ms GAPDH R | ACACGTAACACCAACCTTCC |
